# Settlement dynamics, subsistence economies and climate change during the late Holocene at Nunura Bay (Sechura Desert, Peru): A multiproxy approach

**DOI:** 10.1371/journal.pone.0281545

**Published:** 2023-03-09

**Authors:** Valentina Villa, Nicolas Bermeo, Antoine Zazzo, Christine Lefèvre, Philippe Béarez, Denis Correa, Elise Dufour, Aurélie Manin, Lucie Dausse, Belkys Gutiérrez, Segundo Vásquez, Aurélien Christol, Jean-Jacques Bahain, Nicolas Goepfert

**Affiliations:** 1 CNRS, UMR 7194 Histoire Naturelle de l’Homme Préhistorique (MNHN-CNRS-Université de Perpignan Via Domitia), Paris, France; 2 Université Paris 1 Panthéon-Sorbonne, UMR 8096 Archéologie des Amériques, Paris, France; 3 CNRS, UMR 7209 Archéozoologie et Archéobotanique—Sociétés, Pratiques et Environnements (MNHN-CNRS), Paris, France; 4 Universidad Nacional de Trujillo, Trujillo, Peru; 5 School of Archaeology, University of Oxford, Oxford, United Kingdom; 6 BGL Arqueología, Trujillo, Peru; 7 Université Lyon III Jean Moulin, UMR 5600 Laboratoire Environnement Ville Société, Lyon, France; 8 CNRS, UMR 8096 Archéologie des Amériques (Université Paris 1 Panthéon-Sorbonne), Paris, France; New York State Museum, UNITED STATES

## Abstract

Long considered on the margins, far from the major cultural traditions, the Sechura Desert is situated at the crossroads between the cultures of southern Ecuador and those of the northern Peruvian coast and preserves a large number of varied archaeological sites. Despite this evidence, little is known about the societies that inhabited this region during the Holocene. Exposed to natural hazards, including El Niño events, and to major climatic changes, they were able to adapt and exploit the scarce resources that this extreme environment offered them. Because of this rich history, we have been conducting archaeological research in this region since 2012 in order to clarify the dynamics of human occupation and their links with climate oscillations and environmental changes. This paper present the results of a multidisciplinary study of Huaca Grande, a mound located on Nunura Bay, 300 m from the Pacific Ocean. The nature of the human occupations at Huaca Grande was varied, and several adjustments occurred over time. The subsistence economy was based mainly on local marine resources and a continual use of terrestrial vegetal resources. However, a major change occurred in the more recent occupations, with the apparition of non-local resources (maize and cotton) indicating that Huaca Grande was connected to trade networks. The results show two main phases of occupation separated by a long abandonment (mid-5th century CE to mid-7^th^ century CE and mid-13th century to mid-15th century CE). The occupation of the site appears to have been influenced by changes in the local climate and by extreme El Niño events. Our results highlight the great adaptability of these human groups over the span of a millennium and their capacity to react to the climatic changes and hazards that characterise this region.

## Introduction

**Fig 1:**
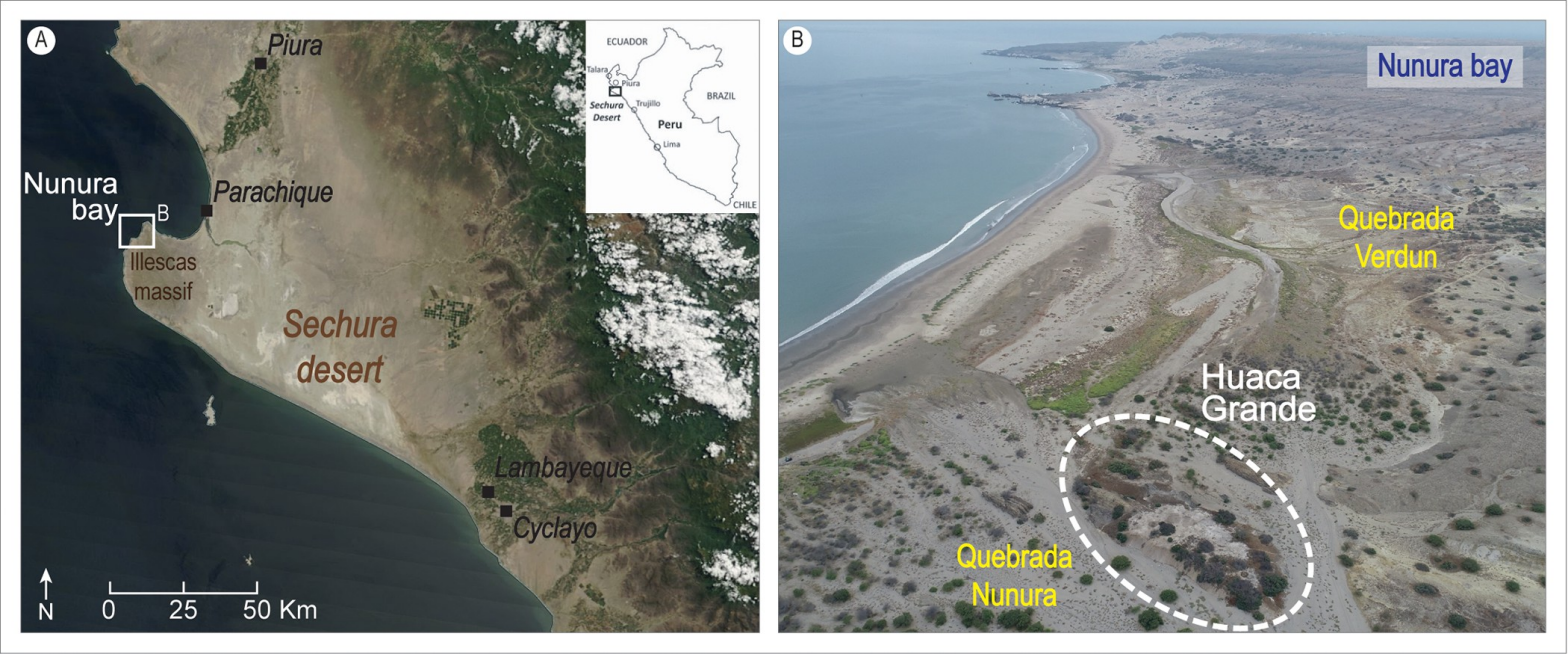
Location of the Huaca Grande archaeological site. A. Location of Nunura Bay and the Illescas massif within the Sechura Desert (satellite photo from NASA Worldview, https://worldview.earthdata.nasa.gov, annotated by the authors). B. Aerial view of Nunura Bay and the Huaca Grande mound (dashed line), showing the location of the two gullies (*quebradas*) near the site.

Despite difficult environmental and climatic conditions, the Sechura Desert has been occupied by human groups for at least 7000 years (from 5590 ± 90 RCY BC) [1–3]. Humans occupied large parts of the desert, but have mainly concentrated around the Illescas massif [4] (Fig 1A). The nature of the archaeological sites differs by period or area, and research in this region has identified shell middens, domestic mounds, specialised fishing sites, civic–ceremonial centres and funerary areas [3–6]. The chronology of these diverse sites is based on ceramic typo-morphological studies and on a total of 30 radiocarbon dates performed on a few of the sites [1, 5, 7]. These data show an intensification of human occupation over time until the period of contact with Europeans.

The coastal environments of northern Peru are particularly sensitive to climatic oscillations and extreme events caused by El Niño (e.g. the Coastal El Niño [also known as El Niño *costero*, or COA EN] of 2017; [7–9]). These changes affect and modify the landscape and the resources available to human groups, forcing them to adapt, to develop new livelihood strategies and sometimes migrate. Therefore, any study aiming to understand the settlement dynamics of this region during the Holocene must necessarily take into account the climatic and environmental variability that marked it throughout this period. Because continental sedimentary archives are rare, incomplete or poorly preserved in the area [10, 11], the archaeological sequences and the evidence they preserve play a fundamental role as environmental and climatic records [e.g. 2, 12–15]. Such data also provide information on the landscapes in which the archaeological sites were located and help researchers to reconstruct environmental change over time, to identify climate forcing and to reconstruct both rapid and long-term changes.

Hence, in this as yet poorly known archaeological and palaeoenvironmental context, for which archaeologists have only fragmentary and incomplete data, we chose to study the Huaca Grande archaeological site (Fig 1B) because of its long and well-preserved stratigraphic sequence that documents a human occupation over the span of a millennium. The site was discovered in the 1970s during the pioneering work of Cárdenas et al. [1, 2] throughout the Sechura Desert. Dating was carried out at the time on two samples, taken at 1.20–1.40 m and 1 m depth, respectively, which yielded uncalibrated dates of 1300 ± 70 RCY BC (PUCP-16) and 1010 ± 80 RCY CE (PUCP-15), respectively [3: 88–85, 89].

We started new excavations there in 2015. Our goal was to achieve a better understanding of human settlement on Nunura Bay (Fig 1) and of the interactions between human societies and the environment over time. This study is part of the researches we are carrying out on a regional scale, on several archaeological sites and on coastal sedimentary archives throughout the Sechura Desert, to reconstruct the cultural evolution of local populations and to understand their adaptation to variations in coastal environments in relation to regional and macro-regional geomorphological and hydro-oceanic-climatic forcings [4, 5, 7, 15, 16].

In order to be able to draw the maximum amount of information from the exceptional archaeological and sedimentary sequence of Huaca Grande, we developed an intra-site multidisciplinary integrated approach that includes geoarchaeological and bioarchaeological studies. This approach, which is still little applied in Peru, is mainly based on detailed and comprehensive chronostratigraphic analyses of the archaeological sequence (micromorphology, sedimentology and geochronology), in combination with the study of the faunal and botanical remains (fish, sea mammals, birds, molluscs, seeds and charcoal) and radiocarbon dating, in order to (i) establish the age and duration of the occupations; (ii) identify the function of the site and the activities that took place there; and (iii) understand the subsistence economies adopted by human groups through the identification of the resources they exploited.

### Geographical and climatic setting of the Sechura Desert

The Sechura Desert is a vast coastal region in northern Peru (lat. 6°S–7°S), situated between the Piura and Lambayeque valleys [17], bordered by the Pacific Ocean to the west and the Andean chain to the east. It is one of the most arid areas in the world, with a climate defined as hot desert according to the classification of Köplen-Geiger [18], determined by the combination of the Peruvian upwelling–Humboldt Current along the Peruvian coast and the rain shadow effect of the Andes [10].

Weather conditions are fairly stable throughout the year. The average temperature is 23.9°C, and the temperature variation between the warmest month (27.1°C, in February) and the coldest month (21.2°C, in August) is about 6°C. The very rare rainfall is concentrated between late summer and early autumn, with a peak of 111 mm on average in March (data from climate-data.org).

The Sechura Desert is located between the 6th and 7th south parallel, in the area where the warm waters of the southward-flowing Ecuador–Peru Coastal Current and the cold waters of the northward-flowing Humboldt–Peru Coastal Current converge [e.g. 19]. From a biogeographical point of view, this area corresponds to the transition between the Panamic (Tropical Eastern Pacific) Province, characterised by tropical, warm-water species, and the Peruvian–Chilean (Warm Temperate South-Eastern Pacific) Province, dominated by temperate-water species [20].

This configuration is affected in a more or less regular way by the activity of El Niño, which influences the atmospheric and/or oceanic circulations in several ways. During Eastern Pacific and El Niño *costero* events, warming of the eastern Pacific Ocean occurs, which results in a deepening of the thermocline and a rise in sea surface temperatures, causing a marked increase in rainfall and resulting flooding on the South-American continent [11 and references therein]. During such events, temperature anomalies in ocean waters are most significant in the area facing the Sechura Desert [21], leading to the southward migration, along the south-eastern Pacific coast, of marine species distributed primarily in the equatorial region [22, 23]. The most severe rainfall events lead to significant changes in coastal environments, e.g. the formation of temporary lakes and lagoons [7, 16, 24], which represent areas of refuge for some of these species and favour their persistence over longer time periods [25, 26].

Continental and oceanic sedimentary archives and archaeological records indicate that the average climate and the activity and intensity of El Niño events were not constant during the Holocene [9, 13, 14, 27–30]. During the past two millennia in particular, three different phases occurred. At the beginning of the common era, a wet phase marked by rare, intense El Niño events is recorded, and this lasted up to 750 CE. Then, between 750 CE and 1250 CE, the average climate became more arid and El Niño was weak. Finally, after 1250 CE, more humid conditions are recorded, together with the most frequent extreme El Niño events [31].

These climatic variations, as well as the impact of El Niño events on coastal environments and on the available natural resources, are key elements that must be taken into consideration in the study and understanding of human settlements in the Sechura region during the Holocene.

### Nunura Bay and Huaca Grande: Background

Huaca Grande is located in the northern sector of the Sechura Desert. It stands in the north-western part of the metamorphic Illescas massif, on the edge of Nunura Bay (Fig 1A). This small inlet, about 3 km wide, is supplied by several *quebradas* (*tr*. gullies), which became filled with water during the more humid climatic phases throughout the Holocene and during El Niño events up to the present day. The two main ones are the Quebrada Verdun, which crosses the central part of the land bordering the bay, running southeast to northwest, and the Quebrada Nunura, which runs along the margins of the Huaca Grande mound and has its mouth to the south of the mound (Fig 1B). Huaca Grande measures 190 m long, 65 m wide, and 7–8 m high and fauna. This mound sits on a rocky hillock approximately 400 m from the Pacific Ocean. The conservation of archaeological remains is good. In fact, shells, bones, charcoal, seeds, ceramics, and metal can be observed on the surface.

During the field campaign by Cárdenas et al. [1, 2] three test pits (labelled Pozo A, Pozo B and Pozo C) and four graves were excavated. Pozo A, whose dimensions were 2 m × 2 m, was quickly transformed into a 12 m × 2 m trench. The graves contained the remains of at least three adults and one child, all associated with funerary goods, including ceramic vessels, necklaces, *Spondylus* valves and metal artefacts, and are attributed to the Chimú culture (3: 79–84). These initial data, in addition to the radiocarbon dating results, encouraged us to resume the excavations on this mound in order to better understand the activities that took place there and to place them in the regional chronological sequence. To do this, we dug two 2 m × 2 m test pits (A4.1 and A4.2)–of which the former was eventually expanded to a 10 m × 5 m area–as well as a 10 m × 5 m excavation area (A4.3). The objective of the latter was to relocate the trench excavated in the 1970s.

## Materials and methods

All the archaeological remains and samples collected during the excavation campaigns are currently stored at the Ministry of Culture of Peru, except for the micromorphological thin sections and the charcoal samples, which are stored at the Malher Centre (Paris, France) and the MSH Mondes (Nanterre, France) respectively.

### Stratigraphy and micromorphology

**Fig 2:**
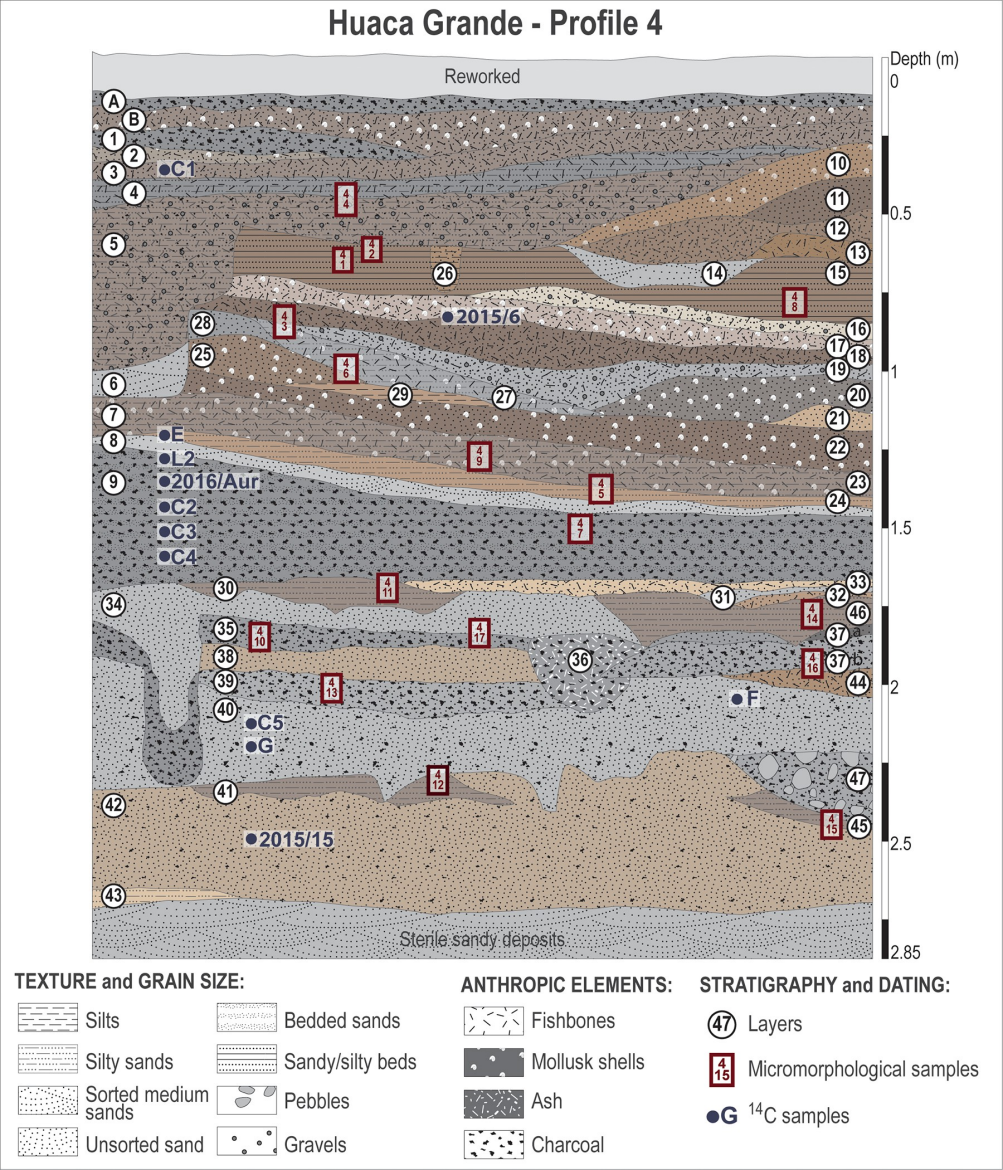
Stratigraphic sequence of profile 4, west wall of trench A4.1, showing the locations from which the micromorphological and radiocarbon dating samples were taken.

Study of the Huaca Grande stratigraphic sequence has required the implementation of a specific methodology adapted to the excavation conditions and the variety of archaeological remains recorded. Stratigraphic profiles 1 (the north wall) and 4 (the west wall) of area A4.1 were systematically described, sampled and studied. Samples of sediment for sedimentological analyses were taken from each layer (Fig 2). In addition, 30 undisturbed sediment samples (about 6 × 9 × 4 cm in size) were collected for thin-section studies from stratigraphic profiles 1, 3 (the south wall) and 4 of the same area to document and study the entire archaeological stratigraphic sequence (see Fig 2 for location of samples along profile 4). Samples were oven dried and impregnated with polyester resin under vacuum. Thin-sections were made at the Servizi per la Geologia laboratory in Piombino, Italy. They were observed under plane-polarised light (PPL) and cross-polarised light (XPL) using a standard Leica DM/LP petrographic microscope. The descriptions follow the guidelines established by Bullock et al. [32] and Stoops [33].

### Chronology

Thirteen samples for ^14^C dating, spanning the entire archaeological sequence, were collected from the stratigraphic profiles and from the camelid deposit found during the excavations (see Fig 2 for location of samples along profile 4). Various materials were AMS radiocarbon dated: camelid faeces (n = 5), charcoal (n = 4), seeds (n = 2) and camelid bone collagen (n = 2): 10 by the ^14^Chrono Centre for Climate, the Environment, and Chronology, The Queen’s University of Belfast (UK); 1 by Beta Analytic (USA); and 2 (the camelid collagen) by AMS *ECHo*Micadas (Laboratoire des Sciences du Climat et l’Environnement [LSCE] Saclay, France), after chemical extraction [34] at the radiocarbon lab at the Muséum national d’histoire naturelle (MNHN, France).

All dates were calibrated at the 2σ age range (≥95%) using OxCal 4.4 [35] and the SHCal20 calibration curve [36]. A Bayesian phase model was built from the available geochronological data using OxCal 4.4, taking into account the stratigraphic constraints; the results of the stratigraphic and micromorphological studies; and the archaeological features, all of which enable the identification of distinct and successive human occupation phases.

### Human remains

The good state of preservation and the representativeness of the excavated skeletons allowed a biological identification of the two individuals buried at Huaca Grande. The estimation of the age at death of these two adult individuals is based on the study of their dentition [37] and the analysis of the sacroiliac surface which allows to refine the age class by chronological intervals with reliability [38]. This probabilistic method is based on the scoring of four morphological characters of the iliac auricular surface: the presence or absence of undulations and striations on the transverse organization (SSPIA), the modification of the articular surface with the progressive appearance of granulation and porosities (SSPIB), the modification of the apical surface with a thin or blunt edge (SSPIC), and the modification of the iliac tuberosity with a smooth or reshaped surface (SSPID).

Regarding sexual diagnosis, the good preservation of the coxal bone of both adult individuals allows the use of two reliable methods based on a world reference sample. The first method is morphoscopic with a minimum reliability of 95% [39]. It consists of assessing five characters distributed over three morpho-functional segments of the bony pelvis: the pre-auricular region, the shape of the greater ischial incisure, the sexual shape of the compound arch, the shape of the inferior border and the relative length of the pubis and ischium. The second method is morphometric based on metric variability with a reliability varying between 98.7% and 100% [40]. It is based on the principle of discriminant analysis from four to ten variables to determine the probability of belonging to a male or female group.

### Fauna

The Huaca Grande faunal remains are comprised of mammals (carnivores, such as pinnipeds and canids, and artiodactyls), birds, fish, molluscs and other marine invertebrates. Each layer of the stratigraphic profile (0–2.80 m) was systematically sampled, and the sediments were dry sieved with a 1 mm mesh sieve. Taxonomic and anatomic identification was carried out using the faunal reference collection available at “BGL Arqueología” lab in Trujillo, Peru, as well as several comparative osteology manuals [41–51].

We used the classical approach of comparative anatomy to estimate the live weight and the length of the fish. The size estimates provided are visual estimates, made by direct comparison with specimens from the fish reference collection of known live weight and length data.

In the case of fish, molluscs and other marine invertebrates, species identification can provide information not only on fishing, collecting and hunting practices, but also on changes in the marine and coastal environments around the site.

### Flora

Archaeobotanical studies were performed on the seeds and charcoal collected during the excavations. Each layer of the stratigraphic profile (0–2.80 m) was systematically sampled for archaeobotanical studies, as were two hearths located in layer 35 (Fig 2). The sediments were dry sieved with a 1 mm mesh sieve. Additionally, six sediment samples of identical volume (10 l) were collected from selected layers rich in organic material (layers 4, 9-U, 9-M, 9-L, 35 and 39), and botanical remains were extracted from them by flotation in order to compare the results with the results derived from dry sieving.

Taxonomic identification of the archaeobotanical remains was carried out using the flora reference collection of “BGL Arqueología” lab; F. Moutarde’s collection of wood charcoal from Peru (archaeobotanical laboratory of the Maison des Sciences de l’Homme [MSH] Mondes, Nanterre, France); A. Chevalier’s Peruvian seed collection (Institut Royal de Sciences Naturelles de Belgique, Brussels); the International Association of Wood Anatomists’ database InsideWood; as well as several publications, atlases and manuals [52–59].

Preliminary sorting allowed the separation of the different types of botanical remains (charcoal, seeds, peduncles, fruits and wood fragments). The carpological remains were observed using a binocular microscope in order to carry out taxonomic identification based on morphological criteria. Charcoal material from each layer was randomly subsampled at least once, using a riffle box. This first subsampling was done in order to be able to export half of each sample and leave the other half in Peru. The charcoal remains were exported under the permits obtained from the Peruvian Ministry of Culture (No 093-2014-VMPCIC-MC) and studied in the archaeobotanical lab of MSH Mondes, where further subsampling was carried out in order to obtain manageable and comparably sized samples for each layer. Charcoal fragments were studied using a reflective light microscope.

## Results

### Sedimentological field description

The Huaca Grande archaeological sequence sits on a sandy substratum, which was reached at a depth of approximately 2.8 m during the archaeological excavations (Area A4.1). Sediments generally have a sandy texture and are extremely rich in organic debris of various kinds (bone, including of fish; marine shell; plant fibre). Layers of mollusc shells and fish remains devoid of matrix alternate with finer, sandy clayey levels that are dark brown in colour and rich in charcoal, burnt seeds and camelid excrement. Whitish powdery layers, composed of ashes, are quite common. Several layers of massive, light grey, sterile sands without any trace of anthropogenic elements have also been identified.

Area A4.1 at Huaca Grande gave the most interesting results, test pit A4.2 having only a short sequence with few materials and Cárdenas’s sequence not having been observed in area A4.3 (S1 Fig). Located at the top of the mound, in its central part, area A4.1 was excavated over four seasons, between 2015 and 2019. We started with a 2 m × 2 m test pit and progressively widened it to a 10 m × 5 m excavation area, with a depth of 2.80 m.

A total of 47 layers were identified within the archaeological sequence of profile 4 (Fig 2; see S1 Table for detailed descriptions of the layers). It can be divided into three parts according to sediment characteristics and the archaeological remains (Fig 2): (1) at the base of the sequence (between −2.70 and −1.66 m in profile 4, layers 43 to 33), are architectural elements, such as walls and post holes, encased in a succession of grey and brown sandy clay deposits rich in charcoal and ashes, alternating with thin layers of light brown clayey silt; (2) in the central part (between −1.66 and −1.45/−1.23 m, layer 9), is a thick, black, sandy silt layer, finely bedded, consisting of an impressive quantity of charcoal, ashes and burnt organic material; (3) at the top (between −1.45 and −1.23 m and the surface, layer 8A), are several stacked, brown and light brown sandy layers rich in molluscs and fish remains, interbedded with levels of ashes and charcoal and several thin, light brown, silty clayey layers.

### Main archaeological features

**Fig 3:**
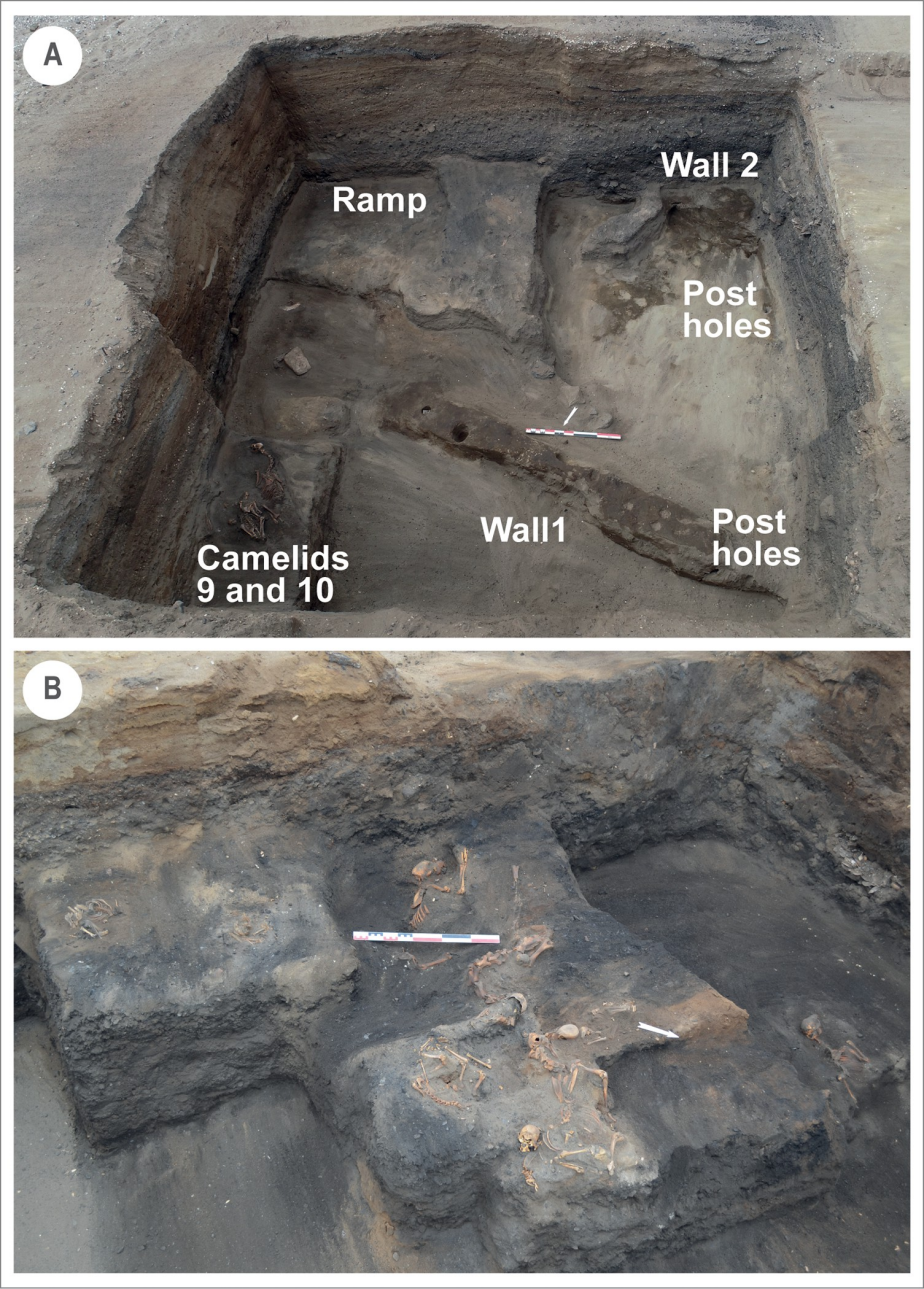
Huaca Grande archaeological features. A. Compact clay structure at the base of the stratigraphic sequence. B. Animal deposit at the top of layer 9.

During the excavations of the Huaca Grande mound, we found multiple levels of cultural occupation (Fig 3A), which yielded some complete and incomplete ceramic vessels. We also observed a deposit of 12 camelids and 9 dogs at the top of layer 9 (Fig 3B). We further observed two 50 cm thick walls of compact clay at 2.20 m depth, at the base of the stratigraphic sequence. These walls, associated with an access ramp and post holes, formed the base of a structure of undetermined function. These architectural features obliged us to adapt the excavation strategy and to gradually widen the initial test pit.

**Fig 4:**
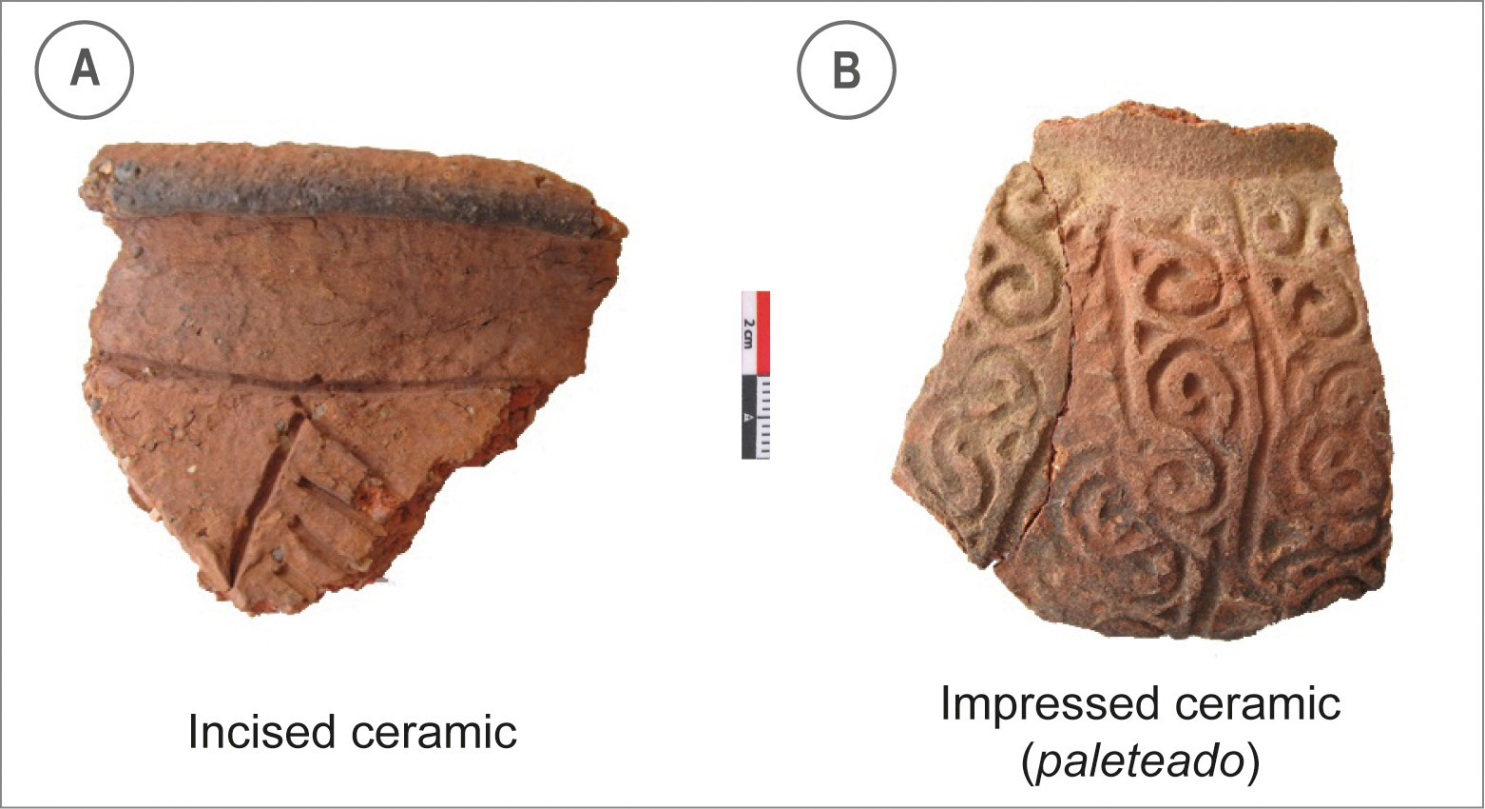
Ceramic shards from area A.41. A. A fragment of incised ceramic, common in the lower part of Huaca Grande archaeological sequence (layers 43 to 33). B. A fragment of *paleteado* ceramic, from the more recent occupation layers of Huaca Grande (layers 8 to A).

Our excavations yielded a total of 168 ceramic shards, belonging principally to small (*olla* and *cántaros*) and large (*tinajas*) jars. At the bottom of the sequence, between layers 43 and 33, we found undecorated and incised ceramics (Fig 4A) which would belong to the Paita B or C phase according to the Lanning sequence [60]. Layer 9 is a transition layer, in which we found both incised ceramics and *paleteado* ceramics, an impressed ceramic type shaped using the paddle-and-anvil technique (Fig 4B). This kind of ceramic becomes predominant in the upper part of the sequence, continuing until the end of the occupation (layers 8 to A).

**Fig 5:**
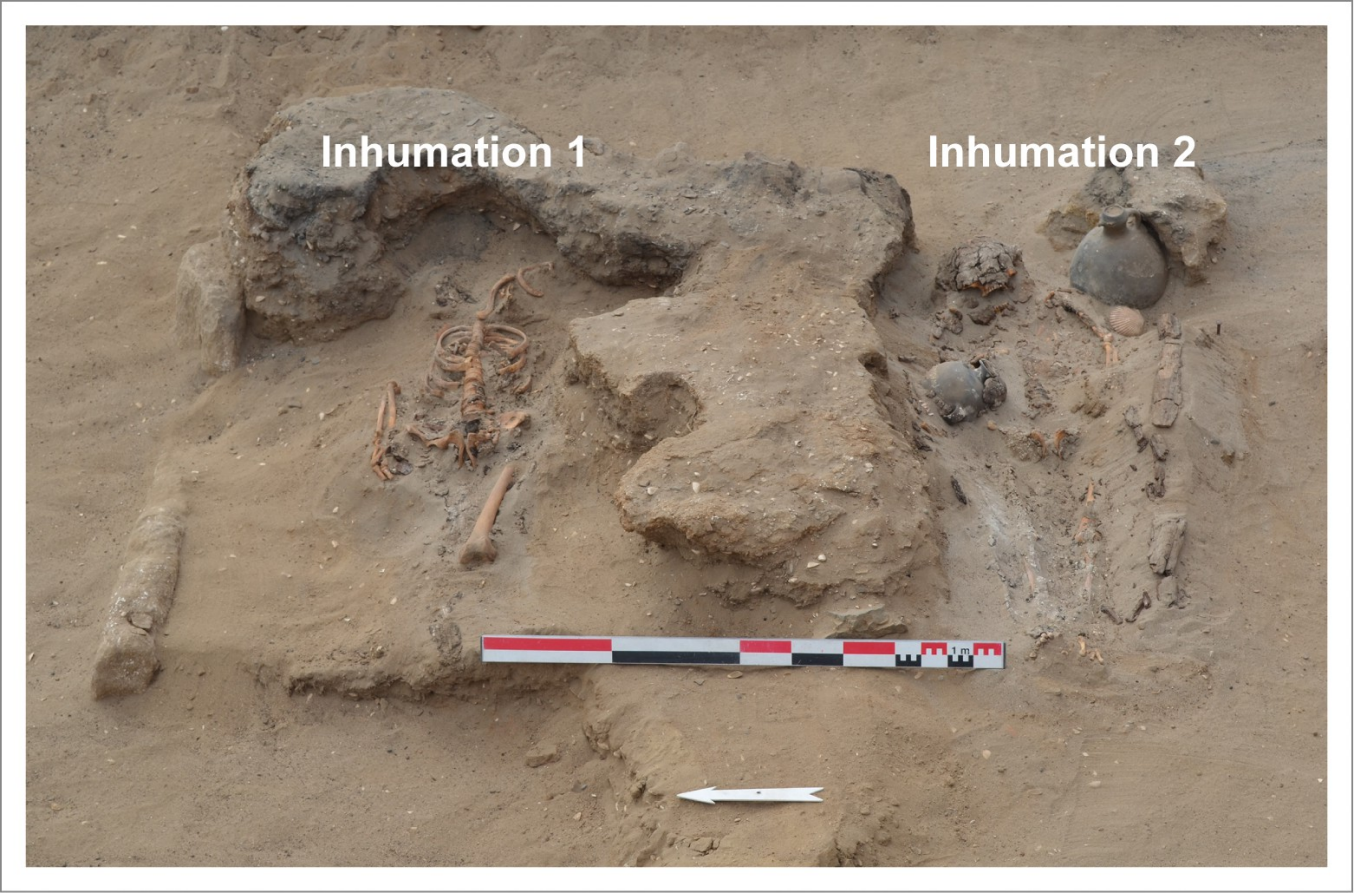
View of the two burials located at the top of the archaeological sequence of area A4.3.

Two human burials were found together at the top of the sequence (layer 5) in excavation area A4.3, located in two pits cutting an earthen soil (Fig 5). They were deposited in dorsal decubitus and oriented east-west with the head to the east. The two individuals are adult males, and are estimated to be about 40 years old and between 20 and 29 years old, respectively. They present a complex mortuary treatment, being wrapped in at least three layers of textile. Individual 2 was accompanied by high-status funerary goods: two ceramics (an *aryballe*, of which the neck was covered by a gourd, and a jar with a bird’s-head-shaped neck), a necklace of shell beads and a bone pendant, two shell bracelets: one made of spondylus (*Spondylus crassisquama*) and the another of an undetermined species, and two scallop half valves (*Argopecten purpuratus*). Ceramics associated with Individual 2 allowed us to determine that this person was affiliated with the Chimú-Inca period (Fig 5).

### Micromorphology

Field observations, in combination with the results of the micromorphological study, made it possible to distinguish 11 sedimentary microfacies (MF) in the Huaca Grande sequence, composed of natural and anthropogenic (occupation) deposits (Table 1). MFs are defined on the basis of several macro- and micromorphological characteristics, such as the nature and texture of the sedimentary matrix; the ratio between elements of natural and anthropic origin; and the nature, arrangement and state of preservation of the macro-remains (bones, molluscs, fish remains, charcoal, ashes and camelid faeces). Each MF can be related to specific activities, sedimentation contexts and anthropic/climatic post-depositional processes [61–64].

**Table 1:** Description of the 11 microfacies identified along the Huaca Grande archaeological sequence.

						Coarse fraction									Fine Fraction	
**MF**	**Microstructure**	**Voids %**	**Types of voids**	**c/f ratio**	**c/f-related distr. pattern**	Aeolian sands	Quartz & mica	Charcoal	Ash	Shells	Fishbones	Excr.	Seeds	Hum. veg. rem.	Colour	B-fabric
**1**	Coarse monic	30	SPV	100/0	Coarse monic	*••••••*	-	*•*	-	*•*	*•*	-	-	-	-	-
**2**	Coarse monic	30	SPV CPV	90/10	Coarse monic	*••••••*	-	*••*	-	*•*	*••*	*•*	-	-	Black	Und.
	Close, fine enaulic				Close, fine enaulic											
**3**	Coarse monic	20	SPV CPV	90/10	Coarse monic	*•••*	-	*•••*	*•*	*•*	*•••*	*••*	-	-	Black/brown	Und.
	Single, spaced enaulic															
					Single, spaced enaulic											
**4**	Close, fine enaulic	30	SPV CPV	70/30	Close, fine enaulic	*••••*	-	*•••*	*•*	*•••*	*•*	*•*	*•*	*•*	Black/yellowish	Und./Cry.
	Single, spaced enaulic															
					Single, spaced enaulic											
**5**	Chitonic	20	SPV CPV	70/30	Chitonic	*••••*	-	*••*	*•*	*•*	*•••*	*•*	*•*	*•*	Black/orange	Und.
**6**	Granular	20	PL VU	50/50	SS/C porphyric	-	*••••*	*•*	-	-	*•*	-	-	-	Yellow	ST-SP
**7**	HS subangular blocky	20	PL	40/60	SS/C porphyric	-	*••••*	-	-	-	-	-	-	-	Orange	ST-SP
**8**	Vesicular	15	VES	70/30	Coarse monic	-	*•••••*	-	-	-	-	-	-	-	Light brown	ST-SP
					Close, fine enaulic											
**9**	Close, fine enaulic	15–20	SPV CPV		SS/C porphyric	-	-	*•••*	*•••*	*••*	*•*	*•*	*•*	-	Black/white	Und./Cry.
**10**	Single, spaced enaulic	20	SPV CPV	70/30	Single, spaced enaulic	*••••*	-	*••••*	*••*	*••*	*••••*	*•*	*•*	*•*	Black/yellowish	Und./Cry.
**11**	Single, spaced enaulic	20	SPV CPV	70/30	Single, spaced enaulic	*•••••*	-	*••••*	*•*	*••*	*••*	*•*	*•*	-	Black	Und.

Type of voids: SPV = simple packing voids; CPV = complex packing voids; PL = planes; VES = vesicles; VU = vughs. C/f ratio = coarse/fine ratio. C/f-related distr. pattern = coarse/fine-related distribution pattern. Coarse fraction: Hum. veg. rem. = humified vegetal remains; Frequencies of coarse fraction components based on the guidelines by Bullock et al. [32]. ·• = very few (<5%); •• = few (5–15%); ••• = common (15–30%); •••• = frequent (30–50%); ••••• = dominant (50–70%); •••••• = very dominant (>70%). B-fabric: Und. = undifferentiated; Cry. = crystallitic; ST-SP = stipple-speckled.

**Fig 6:**
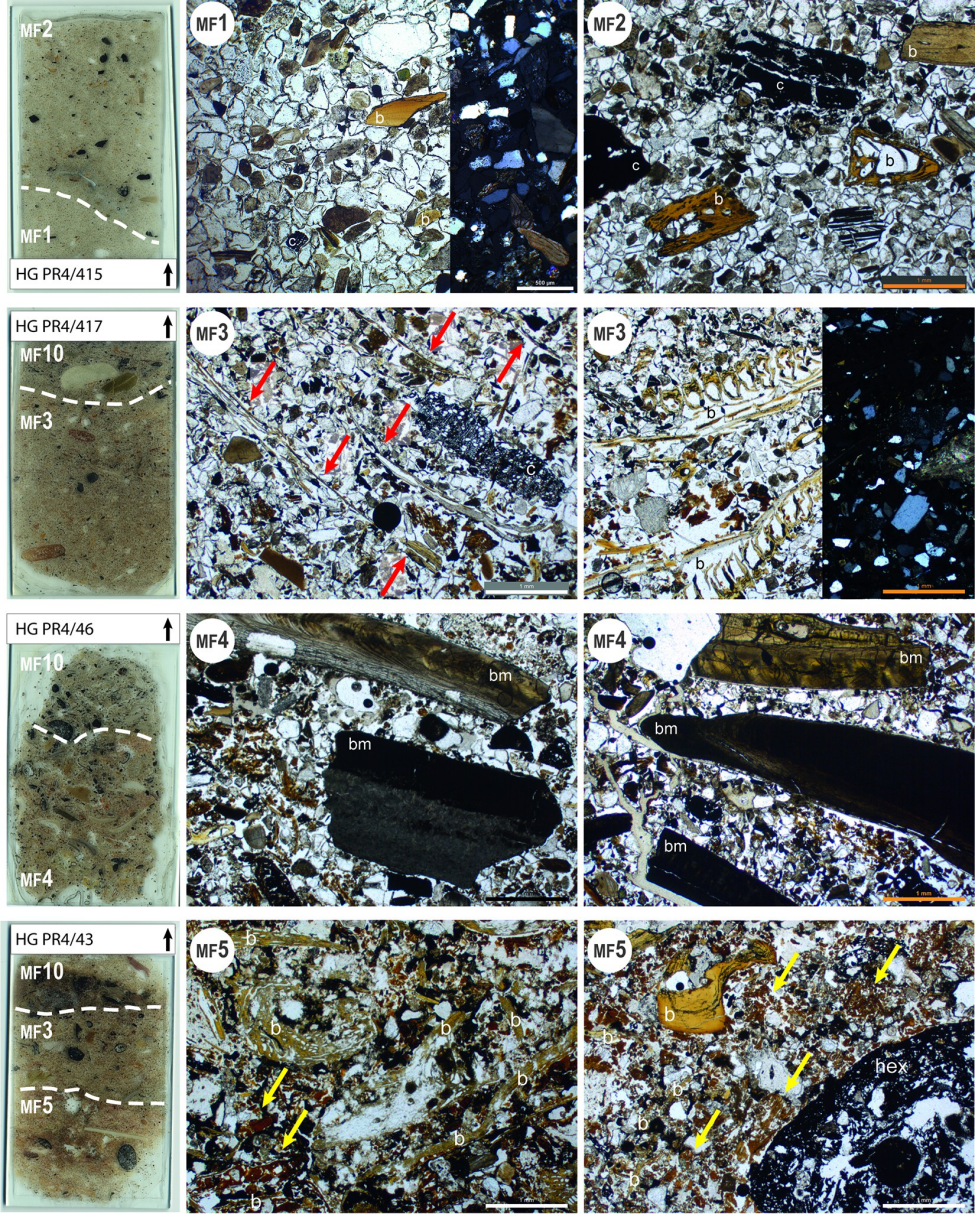
Scans and photomicrographs of thin-sections representing microfacies related to natural (MF1) and occupation (MF2 to MF5) deposits. All images are presented in PPL light. Only the MF1 image and the second of the MF3 images are presented in both PPL (right two thirds of the image) and XPL (left one third of the image). The dashed lines represent the borders between MFs in each thin-section. The main components of the coarse fraction are labelled: b = bones; c = charcoal; bm = burnt mollusc shells; hex = herbivore excrement. Red arrows in the first MF3 photo indicate parallel fishbones. In the second MF3 photo, fishbones are still in anatomical position. Yellow arrows in the MF5 photos indicate aggregates of organic matter, probably remains of fish flesh. Note the higher proportion of aeolian sands (mostly quartz and plagioclase grains) in MF1 and MF2 compared with MF3, MF4 and MF5.

Aeolian sands **(MF1)** are the only natural deposits observed at the site. At Nunura Bay, the beach sands are mostly made up of quartz, plagioclases and limestone grains, as well as some rounded shell fragments. MF1 deposits sometimes contain a few reworked anthropogenic elements relating to the site’s occupation, such as fragments of burnt and unburnt bone, shell and charcoal (Fig 6). These deposits occur at the base and in the middle part of the archaeological sequence of the site (from bottom to top, in layers 43, 42, 8, 6 and 14; Fig 2) and are evidence of periods of site abandonment.

The deposits of anthropic origin, linked to the presence of humans at the site and associated activities, are much more varied. The nature of the main constituents, their abundance, as well as their ratio differ considerably from one layer to another throughout the archaeological sequence (Table 1). This reflects a significant diversification of human activities, exploited resources and functions of the site over time. On this basis, several different categories of microfacies can been distinguished: occupation deposits, earthen floors and combustion features and their waste.

Occupation deposits (**MF2 to MF5**) are mainly composed of burnt and unburnt food waste (bone fragments, shell, gills and other fish remains) and humified organic matter (plant remains and herbivore excrement) mixed with variable amounts of combustion residue (ashes and charcoal) and aeolian sands (Table 1). They testify to a generally domestic occupation (**MF2** and **MF3**), but sometimes traces of activity involving the exploitation of specific resources, in particular molluscs (**MF4**) or fish (**MF5**), can be recognised (Fig 6). The amount of aeolian sands in each layer provides an indication of the rate of accumulation of the occupation deposits and, consequently, of the intensity of site use. A high proportion of aeolian sands (MF2; Fig 6) indicates that some time has elapsed between the different activities, i.e. indicating a rather sporadic human presence at the site. Usually these deposits show no traces of trampling. In contrast, a reduced frequency (MF3**)** or absence (MF4 and MF5; Fig 6) of aeolian sands suggest a more rapid and continuous anthropogenic accumulation.

**Fig 7:**
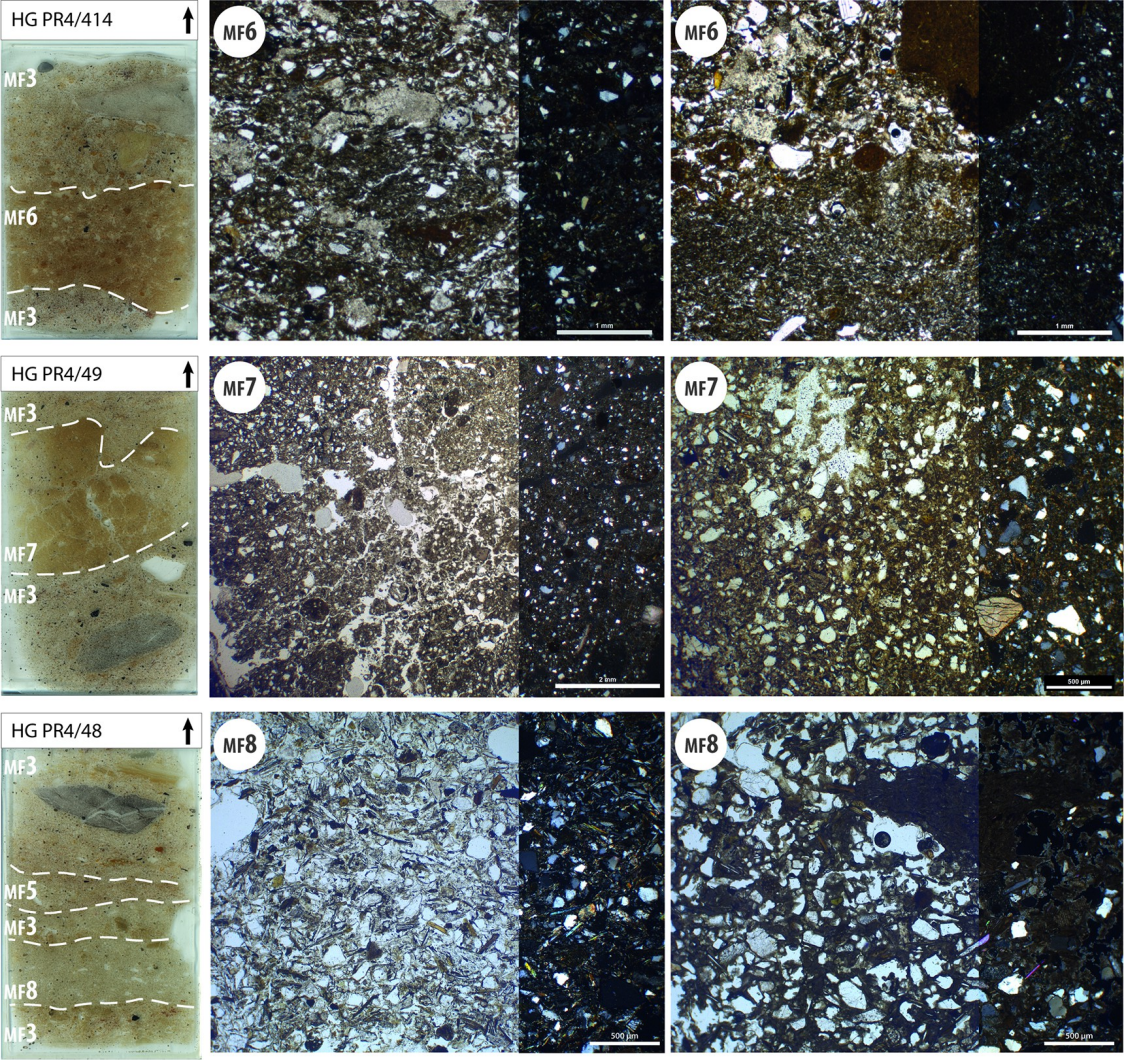
Scans and photomicrographs of thin-sections representing microfacies MF6 to MF8 related to earthen soils. Photomicrographs were taken both in in PPL (right two thirds of the image) and XPL (left one third of the image). The dashed lines represent the borders between MFs in each thin-section. These MFs were distinguished on the basis of the nature of the silty clayey matrix and of the coarse fraction. Note how the proportion of coarse and fine fraction is almost identical in MF6 and MF7, while the coarse fraction (made up of mica, quartz and gypsum grains) is dominant in MF8. MF6 soils show a sub-millimetric, rounded granular structure, while MF7 soils are massive, with larger angular and subangular aggregates produced by trampling and, probably, drying. MF8 soils are thinner, compact (i.e. less porous) and homogeneous.

Some layers attributed to these microfacies are associated with earthen soils (MF6 to MF8; Fig 7). They corresponds to the definition of “active” and “reactive” layers published by Gé et al. [65] and Macphail and Goldberg [66]. They have a clear lower limit and show an abrupt contact with the underlying soils. This succession of earthen soils and occupation layers is particularly common and clearly visible in the upper part of the stratigraphic sequence of Huaca Grande (Fig 2).

Earthen floors (or “clay” floors) (**MF6 to MF8**) are living surfaces intentionally constructed with local soils and/or sediments [67]. They usually show a planar shape, with a sharp upper contact and a gradational, irregular lower one [68]. They can often be found directly superimposed, in sequences alternating with layers of occupation deposits (Fig 7). Reworking, crumbling and inclusion of anthropic materials in the upper part of constructed floors are common, due to trampling.

Three different types of earthen soils were distinguished (MF6, MF7 and MF8), based mainly on the mineralogical nature of their silty clayey matrix. Moreover, observations of thin-sections reveal differences in their microstructure and texture, highlighting that different techniques were used for constructing each of these three types of floors (Fig 7).

Combustion features and waste (**MF9 to MF11**) correspond to deposits and structures containing the physical remnants of fire of anthropogenic origin [69]. They have been classified into three microfacies (Table 1) on the basis of their preservation status: well-preserved burnt residue in original position within domestic fireplaces (**MF9**) and two kinds of reworked residue (**MF10** and **MF11**), the latter two differentiated according to the size of the coarser elements, the size sorting of the burnt residue and the presence or the absence of ash (calcium carbonate and/or calcium oxalate) crystals.

**Fig 8:**
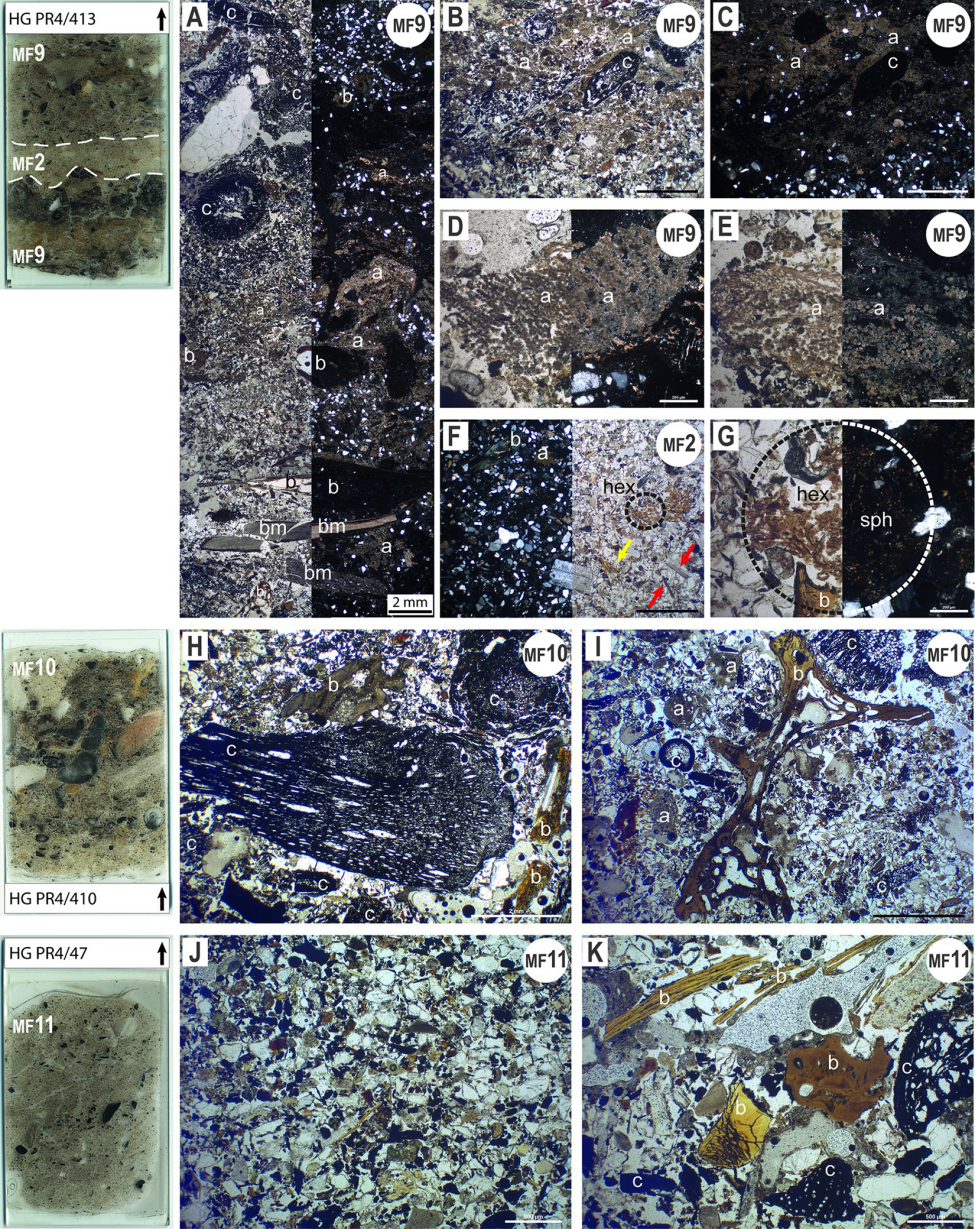
Photomicrographs of thin-sections representing microfacies related to combustion features and waste (MF9 to MF11). **Sample 4/413**: the dashed lines represent the borders between different MF-type layers. MF9 deposits are formed by pairs of stacked, thin, black and yellow layers. Note the layer of occupation deposits (MF2) identified between two distinct burning episodes. **A**: MF9 layers contain charcoal (c), burnt shells (bm) and bones (b) as well as ash crystals (a) (left half in PPL; right half in XPL). **B and C**: In the yellow layer at the base of the sample, ash crystals surround charcoal fragments. Photomicrograph B was taken in PPL while C was taken in XPL. **D and E**: Some burnt vegetal tissues still preserve their original structure (XPL left half; PPL right half). **F**: The MF2-type layer contains some unburnt bone fragments (yellow arrow), mollusc shells (red arrows) and herbivore excrement (dotted circle) (XPL left half; PPL right half). **G**: The same view as F, at higher magnification (PPL left half; XPL right half). Herbivore excrement is easily recognisable in XPL by the presence of spherulites. A fragment of unburnt bone is visible at the bottom of the picture. **H and I**: In the MF10 deposits, large pieces of charcoal and bones are present. Some ash crystal aggregates are also visible (both photos were taken in PPL). **J**: The MF11 layers show a finer texture compared with the MF10 deposits. Aeolian sans are very common, mixed with small (<1 mm) charcoal fragments and food remains (PPL). **K**: The same view as J, at higher magnification (PPL). Some bone and charcoal fragments are visible.

**MF9** layers consist of stacked, thin (about 1 cm thick), horizontal, black (base) and yellow (top) layers bounded by sharp contacts (Fig 8A). The black layers are very dense accumulations of well-preserved charcoal fragments. Some burnt bone fragments and centimetre-sized fragments of mollusc shells are present (Fig 8A). Despite their small size, it is quite easy to recognise that the majority of these fragments are from bivalve shells. In the F9 deposits, the original shape of the shell is often recognisable as having been broken up in situ and the fragments can be seen next to each other (Fig 8A, lower part of the photomicrograph).

The yellow layers consist of calcite ash crystals that surround bone fragments, charcoal microdebris and a few grains of quartz sand (Fig 8B and 8C). The ash crystals are usually chaotically dispersed, but sometimes the original structure of the plant tissues is preserved (Fig 8D and 8E).

These pairs of layers are either continuously stacked or separated by centimetres-thick MF2 layers (Fig 8). Some remains of herbivore excrement are present. They are easily recognisable, as they appear as rounded pellets formed from poorly digested short plant residues and calcitic faecal spherulites (Fig 8F and 8G). Indeed, faecal spherulites are microscopic features (5–15 μm in size) produced in the digestive tract of many animals, especially ruminant herbivores [70 and references therein]. Here, the MF2 layers allow different combustion episodes to be distinguished and indicate a pause (of shorter or longer duration depending on their thickness) in the use of the combustion structures. An interesting fact is that camelid excrement was not used as fuel. It is absent from the burnt layers, whereas it occurs in the intermediate occupation layers.

**MF10** and **MF11** layers result from reworking of combustion residue. The presence of food remains (bones, fishbones, shells and vegetal burnt fragments) throughout these layers suggests that they are mostly made up of material resulting from the maintenance of domestic hearths. According to the experiments performed by Miller et al. [71], the coarser, unsorted and ash-rich deposits that are seen in MF10 (Fig 8H and 8I) could correspond to combustion residue that was scooped up and dumped, probably not far from the structure where it was burnt. On the other hand, the small-sized, sorted and ash-poor deposits of MF11 (Fig 8J and 8K) may result from the sweeping out of domestic hearths. Indeed, in Miller et al.’s experiments, such sweeping out led to textural sorting, with coarser material remaining closer to the hearth’s centre and finer material being scattered farther away [71].

In addition to information on the activities carried out on the site, these deposits also reveal the rhythm of its frequentation. All these massive accumulations of burnt materials of types MF10 and MF11 contain very little aeolian sand. They do not show any discontinuity that could indicate a pause in their formation process. These data therefore suggest that (i) the hearths and combustion structures were cleaned regularly and (ii) the site was stably and continuously occupied at the time.

When the hearts were not emptied and cleaned, deposits of MF9 type were preserved. Covered by layers of aeolian sands (MF1) or by occupation layers (MF2-type), these deposits attest to the last phase of use of the hearths, either before a period of abandonment or before a new organisation of the site where the combustion structures were located elsewhere or were absent.

### ^14^C dating and Bayesian analysis

The 13 radiocarbon dates provide a coherent set, outlining a robust chronological framework for the Huaca Grande archaeological sequence (Table 2).

**Table 2:** Radiocarbon sample details and results.

AMS lab number	Sample	Layer	Depth(cm)	Type of material	C/N	Collagen yield %	Radiocarbon age (BP)		Calibrated age range (cal CE) (2σ)	
							Date	Error	From	To
UBA-35563	C1	3	36	Seeds			545	30	1399	1450
UBA-30843	2015/6	18	83	Camelid faeces			795	30	1222	1292
UBA-41156	E	24	120	Camelid faeces			755	20	1270	1383
ECHo 2998.1.1	HGC-10	8/9	128	Bone collagen	3.2	15.2	1150	25	891	1012
ECHo 2999.1.1	HGC-4	8/9	128	Bone collagen	3.2	12.7	1145	25	892	1014
Beta-454075	2016/Aur	9	135	Charcoal			1430	30	599	768
UBA-35564	C2	9	144	Charcoal			1420	30	602	770
UBA-35565	C3	9	152	Camelid faeces			1465	25	590	659
UBA-35566	C4	9	159	Seeds			1505	30	545	645
UBA-41157	F	40	205	Charcoal			1470	25	588	657
UBA-35567	C5	40	213	Charcoal			1545	25	479	639
UBA-41158	G	40	220	Camelid faeces			1545	20	525	637
UBA-30844	2015/15	42	249	Camelid faeces			1600	30	421	578

Unmodelled data (95.4% probability) and data calibrated using Oxcal 4.4 and the SHCal20 calibration curve [35, 36]. UBA = The Queen’s University of Belfast; ECHo = ECHo-Micadas; Beta = Beta Analytic.

**Fig 9:**
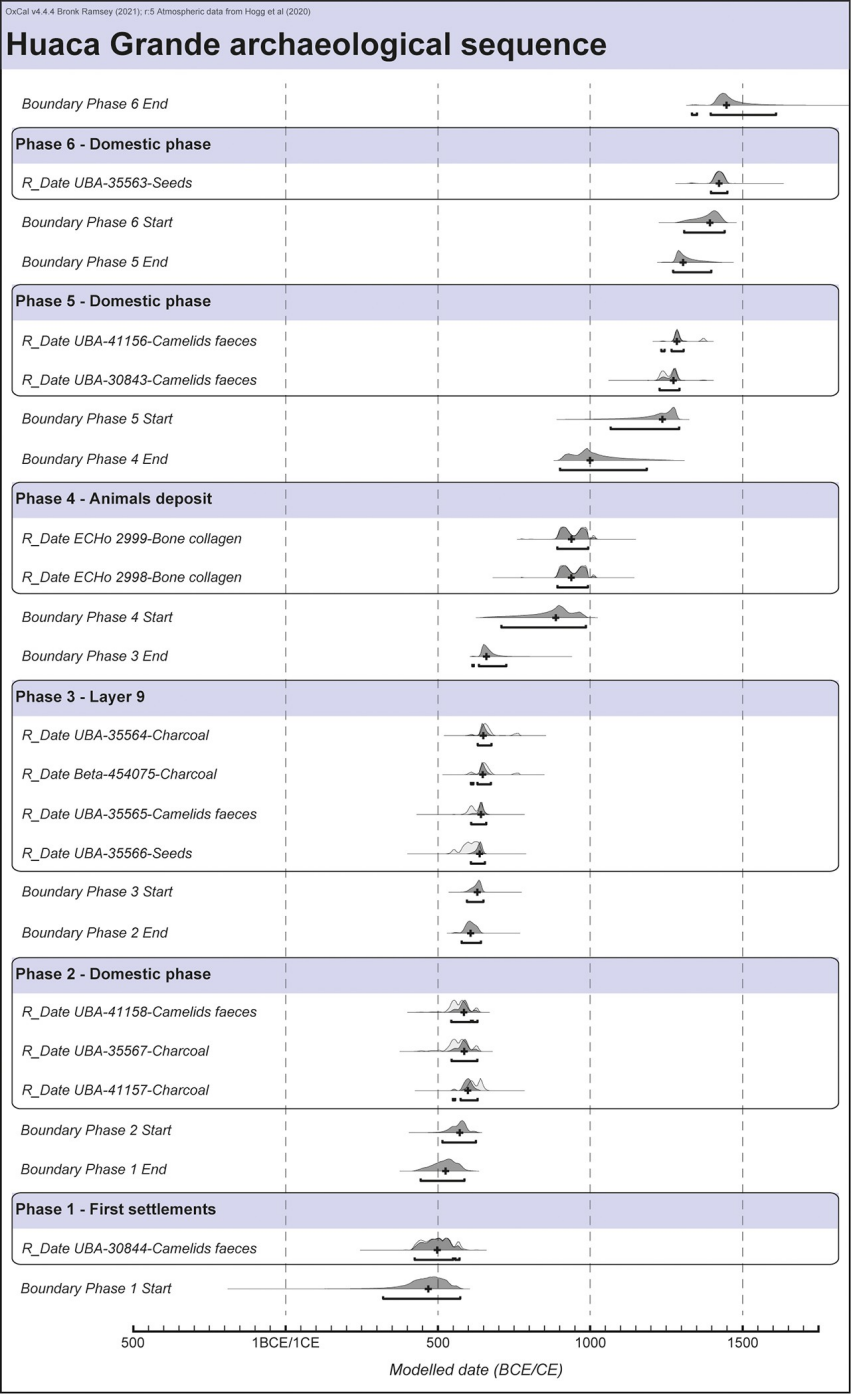
Bayesian chronological modelling of radiocarbon ages from the occupation phases of the Huaca Grande archaeological site. The simple calibrated radiocarbon age distributions are represented in light grey; the posterior probability distributions determined via the modelling are represented in dark grey. This figure was generated using OxCal 4.0 [35] and then modified.

A Bayesian model was created from these dates, based on the archaeological and stratigraphic data collected during the excavations and on the results of the micromorphological study (Fig 9). Changes in the structure and function of the site and periods of abandonment revealed by layers of sterile aeolian sands were used as benchmarks to establish the beginning and end of the phases of human occupation (“boundaries” in Fig 9 and Table 3) and to constrain the model (see S1 Appendix for the OxCal codes of the model).

**Table 3:** Modelled start and end date chronology of the six main occupation phases identified in the Huaca Grande archaeological sequence.

		Modelled date (cal CE)			Duration
		**From**	**To**	**Median**	**(median)**
**Phase 6**	Boundary Phase 6 end	1334	1610	1448	55
**Domestic phase**	Boundary Phase 6 start	1309	1441	1393	
	Interval between Phase 5 and Phase 6	0	136	70	
**Phase 5**	Boundary Phase 5 end	1273	1398	1305	68
**Domestic phase**	Boundary Phase 5 start	1067	1292	1237	
	Interval between Phase 4 and Phase 5	17	350	215	
**Phase 4**	Boundary Phase 4 end	901	1186	1000	113
**Animals deposit**	Boundary Phase 4 start	709	986	887	
	Interval between Phase 3 and Phase 4	37	329	220	
**Phase 3**	Boundary Phase 3 end	614	725	660	30
**Layer 9**	Boundary Phase 3 start	596	650	630	
	Interval between Phase 2 and Phase 3	0	49	16	
**Phase 2**	Boundary Phase 2 end	578	642	608	36
**Domestic phase**	Boundary Phase 2 start	515	625	572	
	Interval between Phase 1 and Phase 2	0	117	39	
**Phase 1**	Boundary Phase 1 end	444	588	526	57
**First settlements**	Boundary Phase 1 start	321	574	469	

The resulting model is very robust, as shown by the agreement index (Amodel = 100.5; Aoverall = 103.4), and does not include any outliers. Six main occupation phases and some abandonment periods can be distinguished. Taking into account the median duration calculated from the modelled data (Table 3), the duration of occupation varies between 30 years (phase 3 –Layer 9) and 120 years (phase 4 –animal burial) and that of abandonment varies between 16 years (between phase 2 and phase 3) and 220 years (between phase 3 and phase 4).

In summary, the chronological data show that the first human settlement started early in the second half of the 5th century CE (469 cal CE). The site was then occupied, with one or two brief interruptions, until the second half of the 7th century CE (660 cal CE), as attested by the dense succession of combustion structures, occupation layers and accumulations of burnt residue (phases 1, 2 and 3).

The deposit of complete camelids and dogs, located between layer 9 and layer 8 (phase 4) and dated between the end of the 9th century and the beginning of the 11th century CE, would have been preceded (after the end of phase 3 –layer 9) and followed (before the beginning of a new domestic occupation–phase 5) by periods of abandonment, each one lasting just over two centuries (Table 3). These data need to be discussed for further precision. Indeed, they are biased by the calibration results of the two dates on the camelid remains, which spread them over more than a century (between 887 and 1000 cal CE). However, we know from the excavation observations that the deposit of the animals occurred in a single episode that was of extremely short duration. It can therefore be considered an isolated action that briefly interrupts a long phase of abandonment that lasted about six centuries (between the end of the formation of layer 9 and the beginning of phase 5).

Domestic human occupation of the site did not recommence until the middle of the 13th century CE, after which it continued until about the middle of the 15th century CE, marking the last human occupation of the site (Fig 9). The model confirms the presence of an abandonment period between phase 5 and phase 6, as suggested by the presence of the aeolian sandy deposits of layers 14 and 6. This period would have lasted about 70 years, covering most of the 14th century CE (Table 3).

### Zooarchaeological analyses

#### Malacofauna and other marine invertebrates

**Fig 10:**
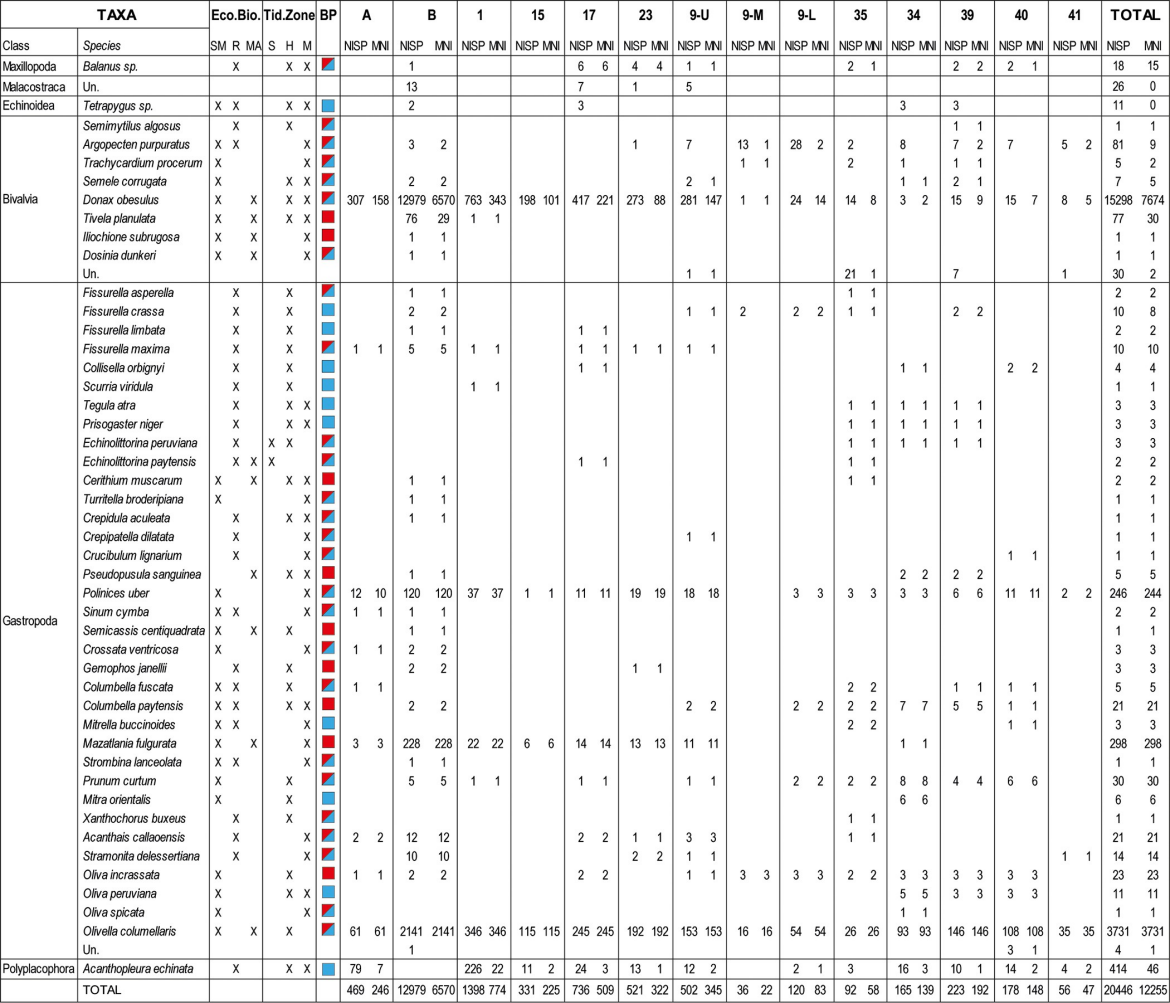
Huaca Grande molluscan assemblage: Ecological biotope, vertical distribution, geographic distribution, NISP and MNI of the taxa identified in trench A4.1.

We analysed 20,446 malacological remains (number of identified specimens, NISP) from Huaca Grande, corresponding to a total weight of 26 kg and representing a minimum number of individuals (MNI) of 12,255 (Fig 10). The level of detail of the taxonomic identifications varies: we were able to identify to the species level for 44 species, the genus level for 2 taxa, and the class level for 3 taxa.

A red square indicates that the taxon lives in the Panamic Province and in the Paita Transition Zone; a blue square indicates that it lives in the Peruvian–Chilean Province. Eco. Bio. = ecological biotope; SM = sandy-muddy; R = rocky; MA = mangrove; Tid. Zone = tidal zone; S = spray zone; H = high-tide zone; M = middle tide zone; Un. = undetermined.

Despite the fact that many species of molluscs need specific environmental conditions to thrive, some taxa can occur in more than one biotope [41, 72]. Thus, some species were attributed to mixed biotopes, consisting of two different biotopes (Fig 10). The ecological zone, corresponding to the vertical and geographical distribution in which the different identified species develop, was determined. Three ecological biotopes are represented in the Huaca Grande faunal record: sandy-muddy, rocky, and mangrove. For each biotope, the spray, high tide and middle tide zones were distinguished (Fig 10). Similarly, the distribution area of the identified species was documented, with all of them falling either within one of the two biogeographic provinces (Panamic or Peruvian–Chilean) or in the transition zone between them (Paita Transition Zone; Fig 10).

Mollusc remains are abundant in the Huaca Grande sequence, but their distribution within the stratigraphic sequence is discontinuous. They occur in layers A, B, 1, 15, 18, 8, 9 (upper, middle and lower), 35, 34, 39, 40 and 41. They are very rare in the oldest layers and become very common in the upper part of the sequence, which corresponds to the most recent occupations. The combined NISP of layers A, B and 1 is 14,846 (representing 72.6% of the total; Fig 10).

The greatest diversity was found in layer B, where 29 species were identified, and in layers 35, 34 and 39, each yielding 20 species. The most common species in the malacological assemblage are *Donax obesulus*, with 15,298 NISP (74.8%); *Olivella columellaris*, with 3,731 NISP (18.2%); *Acanthopleura echinata*, with 414 NISP (2%); and *Mazatlania fulgurata*, with 298 NISP (1.5%) (Fig 10). The most common taxa exploited for consumption were *Donax obesulus*, *Acanthopleura echinata* and *Argopecten purpuratus*, with a combined total of 15,793 remains (representing 77.2% of the total) and an MNI of 7,729.

### Fish

A preliminary study of ca. 80% of the fish remains that came from area A4.1 permitted us to recognise a rather high diversity of species, mostly characteristic of the modern temperate environment of the Illescas Peninsula. The NISP is 1,332. Sardines (Clupeidae) are the dominant taxon (Table 4). They are followed in terms of NISP by a fairly good number of cartilaginous fishes, both sharks (Selachii) and rays (Batoidea), and by chub mackerel (*Scomber japonicus*; Scombridae). Croakers (Sciaenidae) show the greatest diversity, being represented by at least five species. Also noteworthy is the good representation of the wrasses (Labridae), which are relatively rare in Peruvian archaeological sites [73].

**Table 4:** Huaca Grande fish assemblage. NISP and proportions of the taxa identified in trench A4.1.

Taxon	NISP	%
Clupeidae (sardines)	659	49.47
Batoidea (skates and rays)	188	14.11
Selachii (sharks)	152	11.41
Scombridae (mackerels)	144	10.81
Sciaenidae (croakers)	67	5.03
Labridae (wrasses)	50	3.75
Carangidae (jacks)	21	1.58
Muraenidae (moray eels)	19	1.43
Merlucciidae (hakes)	7	0.53
Triglidae (searobins)	7	0.53
Haemulidae (grunts)	6	0.45
Mugilidae (mullets)	5	0.38
Labrisomidae (blennies)	2	0.15
Serranidae (sea basses)	1	0.08
Ophidiidae (cusk-eels)	1	0.08
Ariidae (sea catfishes)	1	0.08
Centrolophidae (medusafishes)	1	0.08
Nematistiidae (roosterfishes)	1	0.08
**Total**	**1332**	**100**

Area A4.1 is characterised by a fairly high proportion of sardines, guitar rays (Rhinobatidae and Batoidea) and sharks (Selachii). This could reflect active fishing with nets, either gillnets or seines, in the sandy bay in front of the site. However, angling was also practised, at least on rocky substrates, as evidenced by the presence of benthic and demersal species, such as moray eels (Muraenidae) and wrasses and the hooks found within the archaeological sequence of the site. Most of the other taxa identified are poorly represented and are essentially demersal taxa: e.g. *Merluccius gayi* (Merlucciidae), *Prionotus stephanophrys* (Triglidae) and grunts (Haemulidae). This faunal spectrum seems very close to what could be found along the Illescas Peninsula today.

As far as the sizes of the fish caught are concerned, they are generally small or medium throughout the entire sequence of trench A4.1. Layer 18 is the richest in fish remains and in particular in remains of small pelagic fish, such as sardines and mackerels.

### Mammals and birds

The mammal remains from Huaca Grande were separated into two sets: the first comprises the scattered remains of Cetacea, Carnivora, Artiodactyla and Rodentia found in several layers across the stratigraphic sequence, and the second comprises a ritual deposit of camelids and dogs stratigraphically located between layers 9 and 8.

We found 1,601 mammal remains, including 1,325 fragments (82.8% of the NISP) smaller than 5 cm, which corresponds to the total assemblage. They are unevenly distributed along the stratigraphic sequence (Table 5), occurring in layers B, 1 and 18 and in layers 8 to 41 (and with more frequency in layers 8 to 39). Otariidae are the most frequent mammals recorded, with 213 remains (13.3% of the total), and their remains occur with more frequency in the bottom of the sequence, in particular in layers 34 to 9-L. Both South American sea lion (*Otaria byronia*) and South American fur seal (*Artocephalus australis*) have been identified in the assemblage, the latter being represented by at least four remains. The other taxa are rarer: Camelidae (n = 48), dogs (*Canis familiaris*, n = 11), Cetacea (n = 3) and Rodentia (n = 1).

**Table 5:** Huaca Grande mammalian and avian assemblages: NISP of the taxa identified in trench A4.1.

Mammalia			A	B	1	15	16	18	19	8	9-U	9-M	9-L	35	34	39	40	41	Total NISP
Cetacea	Undetermined													3					3
Carnivora	Canidae	*Canis familiaris*						5						1	4		1		11
	Otariidae	*Otaria byronia*		2	4			27		3	12	9	33	71	31	8	8	5	213
Artiodactyla	Camelidae	Camelidae sp.		23	12			11				1					1		48
Rodentia														1					1
Undetermined (fragment < 5 × 5 cm)					4			299		108	155	124	265	204	84	80	2		1325
		**Total Mammalia**	**0**	**25**	**20**	**0**	**0**	**342**	**0**	**111**	**167**	**134**	**298**	**280**	**119**	**88**	**12**	**5**	**1601**
**Aves**																			
Sphenisciformes	Spheniscidae	*Spheniscus humboldti*															1		1
Pelecaniformes	Pelecanidae	*Pelecanus thagus*											1	2					3
Suliformes	Phalacrocoracidae	*Phalacrocorax bougainvilliorum*		2	1	1		28			1		1	3	2			2	41
		*Phalacrocorax* cf *brasilianus*						2		1							3		6
		*Phalacrocorax* sp.															1		1
	Sulidae	*Sula variegata*						4				1		1		4	2		12
	Undetermined	Undetermined								1									1
Charadriiformes	Laridae	*Larus* cf *pipixcan*		1							1		2	1		1			6
Columbiformes	Columbidae	*Columba* cf *modesta*									4				1				5
Undetermined								1			1				1				3
		Total Aves	0	3	1	1	0	35	0	2	7	1	4	7	4	5	7	2	79
		**Total Mammalia + Aves**	**0**	**28**	**21**	**1**	**0**	**377**	**0**	**113**	**174**	**135**	**302**	**287**	**123**	**93**	**19**	**7**	**1680**

The presence of the remains of 12 camelids and 9 dogs in a pit dug into the top of layer 9 was unexpected. Camelids were found with more frequency in the eastern part of the excavation, while the dogs were recorded in the western part. The specimens were buried either complete or incomplete (without the head), and no specific position or orientation were observed. All the camelids were juveniles, with a biological age estimated from new-born to 9 months. Apart from one adult, all the dogs were juveniles less than 12 months old. No cutmarks were registered neither on camelids and dogs.

Bird remains are uncommon throughout the sequence. We found only 79 remains (NISP), corresponding to a total weight of 700 g (Table 5). Their distribution within the stratigraphic sequence is continuous, and they occur in layers B, 1, 15, 18 and 8 to 41, with 35 remains in layer 18 (44% of the total). Cormorants are the most numerous (n = 48), with Guanay cormorant (*Phalacrocorax bougainvilliorum*, n = 41) and, possibly, neotropic cormorant (*Phalacrocorax brasilianus*, n = 6), plus one *Phalacrocorax* specimen not attributed to species. The second-most important species is Peruvian booby (*Sula variegate*, n = 12). Note also the presence of Franklin’s gull (*Larus pipixcan*, n = 6), turtledove (*Columba modesta*, n = 5), Peruvian pelican (*Pelecanus thagus*, n = 3), and, rarer, Humboldt penguin (*Spheniscus humboldti*, n = 1). Four bird remains were undetermined.

### Archaeobotanical analyses

#### Anthracology

**Fig 11:**
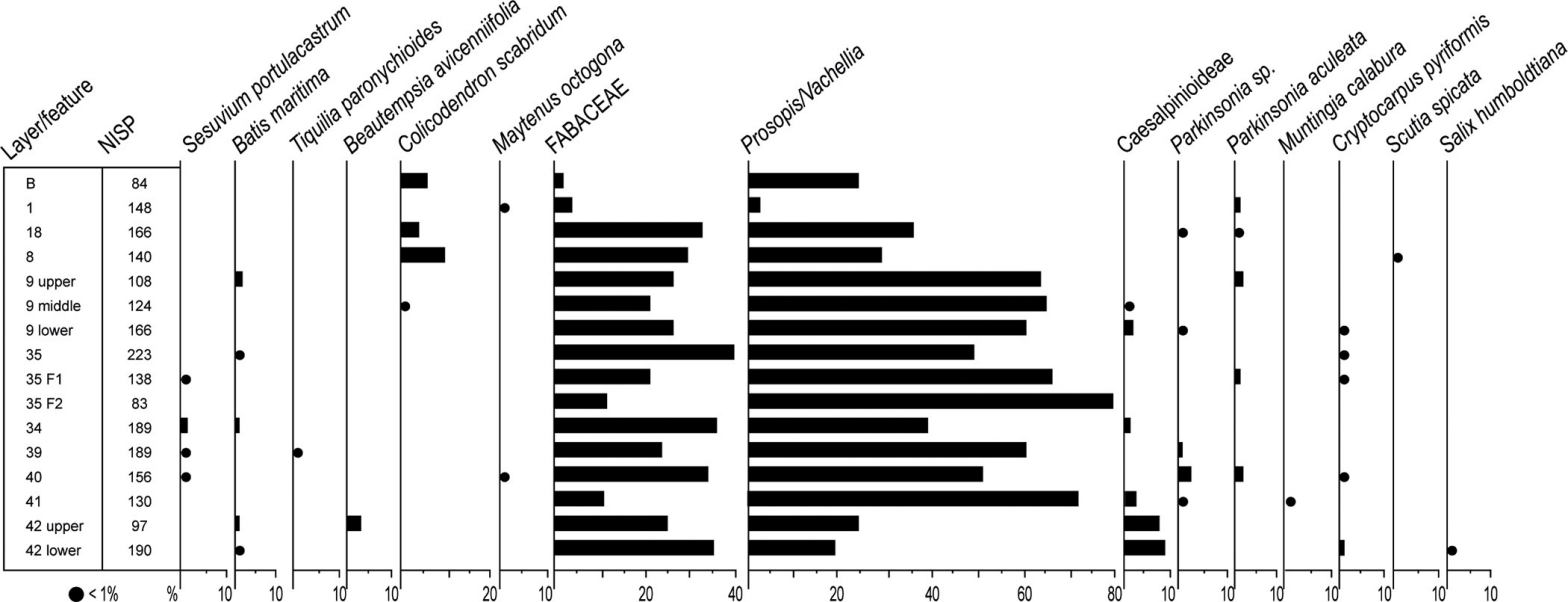
Huaca Grande anthracological assemblage. NISP and proportions of the taxa identified in trench A4.1.

A total of 2,331 pieces of charcoal were recovered and analysed; 1,768 (75.8%) of them were identified to varying taxonomic ranks and assigned to 1 family, 1 subfamily, 1 genus, 1 genus group and 11 species. The number of taxa varies throughout the sequence (Fig 11). Layers 40 and 42-L present the highest number of identified taxa (7 and 6, respectively), feature 35-F2 and layer B present the lowest (2 and 3, respectively). All other layers present at least 4 or 5 different taxa. This variability results in part from the different sample sizes (number of studied charcoal fragments). For example, the samples with the lowest taxonomic diversity (B and 35-F2) are indeed the smallest in term of NISP. However, sample size is not the only factor, as layer 40, presenting the highest diversity, has an average sample size (NISP = 156), and layer 35, with the largest sample size (NISP = 223), presents an average diversity.

The anthracological spectrum (Fig 11) is dominated by the family Fabaceae and particularly by the genus group *Prosopis/Vachellia* [52, 74–76]. In the area and close to Huaca Grande, the genus group may be represented by *Prosopis* sp. (american carob) called *algarrobo* in Spanish, *Vachellia macracantha* (longspine acacia), known locally as *faique* and *Vachellia aroma* (aromatic acacia) var. *huarango* called *espino*. They are the most ubiquitous taxa, and they are the only ones present in every layer/feature of the stratigraphic sequence. The second-most ubiquitous taxon is the subfamily of the Caesalpinioideae, also family Fabaceae, present in the lower (layers 41 to 42-L) and middle (layers 9-M, 9-L and 34) parts of the sequence. *Parkinsonia* sp. and *Parkinsonia aculeata* (Jerusalem thorn known in Spanish as *espino de Jerusalem*) belong to this subfamily and together are the next-most ubiquitous taxa, along with *Batis maritima* (saltwort called *vidrio* in Spanish) and *Cryptocarpus pyriformis* (salt bush). Other notable taxa, present in one quarter of the samples, are S*esuvium portulacastrum* (sea purslane), present in the lower half of the sequence (layers 35-F1 and 34 to 40) and *Colicodendron scabridum* (scabrid colicodendron known locally as *sapote*), present in the upper half of the sequence, particularly in the most recent layers (layers B, 18, 8 and 9-M). Other taxa are sporadic, occurring mostly in only a single sample (Fig 11).

The Fabaceae also comprise the largest number of specimens, representing 59% to 91.6% of the analysed charcoal in all layers except for the upper ones (B and 1), where they represent only 26% and 7.4% of the samples, respectively. The genus group *Prosopis/Vachellia* is the most abundant in terms of fragment counts (2%–79.5%). Caesalpinioideae percentages are much smaller even when we include the sub-taxa (1%–4%). Among all other single taxa, only *C*. *scabridum* is appreciably present (3.6%–9%) in layers A, 18 and 8.

### Carpology

We analysed a total of 20,065 carpological remains (NISP), i.e. seeds and fruits and fragments thereof. Of these, 1,397 were recovered from dry-sieved samples (7% of the total) and 18,668 from flotation samples (93%). A total of 16,680 remains were identified taxonomically (83%), representing 13 different taxa: 3 genus and 10 species. These percentages show that the two recovery methods led to very different results in the number of collected botanical remains. The flotation method allowed us to retrieve remains smaller than 1 mm and, therefore, to identify a different set of taxa compared with the dry sieving method.

**Fig 12:**
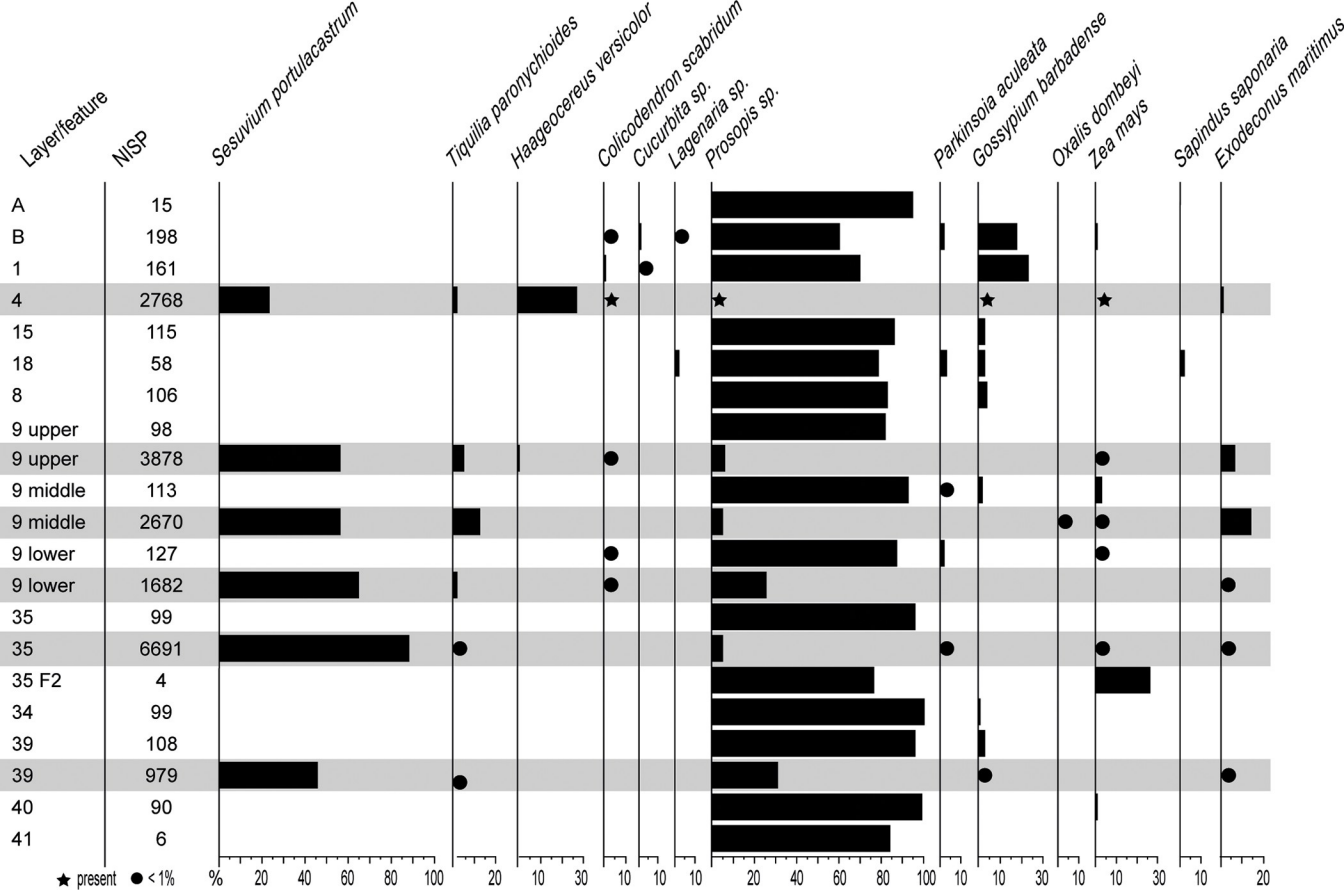
Huaca Grande carpological assemblage. NISP, ubiquity and/or proportions of the taxa identified in trench A4.1. Grey shading highlights flotation samples.

Taxonomic diversity is low in most layers, with a tendency of richer layers towards the middle and upper parts of the sequence: layer B presents seven identified taxa, followed by layer 18, with five taxa (Fig 12). Only one taxon (*Prosopis* sp.) occurs in layers 41, 35, 9-M and A, and only one feature, 35-F1, did not yield any carpological remains.

Also in the carpological remains, the Huaca Grande sequence is characterised by the ubiquitous presence of Fabaceae, and more specifically by the carob tree (*Prosopis* sp.). This taxon is dominant in all the dry-sieved samples, ranging from 99% to 60% of NISP. The second-most ubiquitous taxon is cotton (*Gossypium barbadense*). It occurs in nine layers, and it is the second-most abundant taxon in terms of NISP, ranging from 1% to 23%. It is unequally present in the lower (layers 34 and 39) and middle (layer 9-M) parts of the sequence, and it is much more frequent, both in ubiquity and in quantity, in the upper part (layers B to 8; Fig 12).

Maize (*Zea mays*) is present in seven samples, mostly in layer 9 (samples 9-U, 9-M, and 9-L) and to a lesser extent in the lower part of the sequence (layers 35 and 40). It has a lower count than *Parkinsonia aculeata*, which occurs in five layers, in the middle (layers 9-M to 35) and upper (layers B and 18) parts of the sequence. *Colicodendron scabridum* is also present in five different samples, corresponding to layer 9 (9-U and 9-L) and to the upper part of the sequence.

Squash (*Cucurbita* sp.) and gourd (*Lagenaria* sp.) are present in two layers each in the top of the sequence (layers B and 1 and layers B and 18, respectively). Soapberry (*Sapindus saponaria*) occurs only in layer 6.

All other identified taxa were recovered only by flotation (Fig 12). These samples provided most of the taxa already found in the dry-sieved samples, except for the two Cucurbitaceae and *S*. *saponaria*. Among the additional taxa, *Sesuvium portulacastrum*, *Tiquilia paronychioides* and *Exodeconus maritimus* are present in all of the flotation samples. The first is by far the most frequent in each sample, especially in layers 35 and 9 (9-U, 9-M and 9-L). The other two show comparable proportions and are more frequent in layer 9 (9-U and 9-M). *Haageocereus versicolor* occurs only in layers 9-U and 4 and is particularly frequent in the latter. *Oxalis dombeyi* is present only in layer 9-M.

## Discussion

### Site occupation and subsistence economies

**Fig 13:**
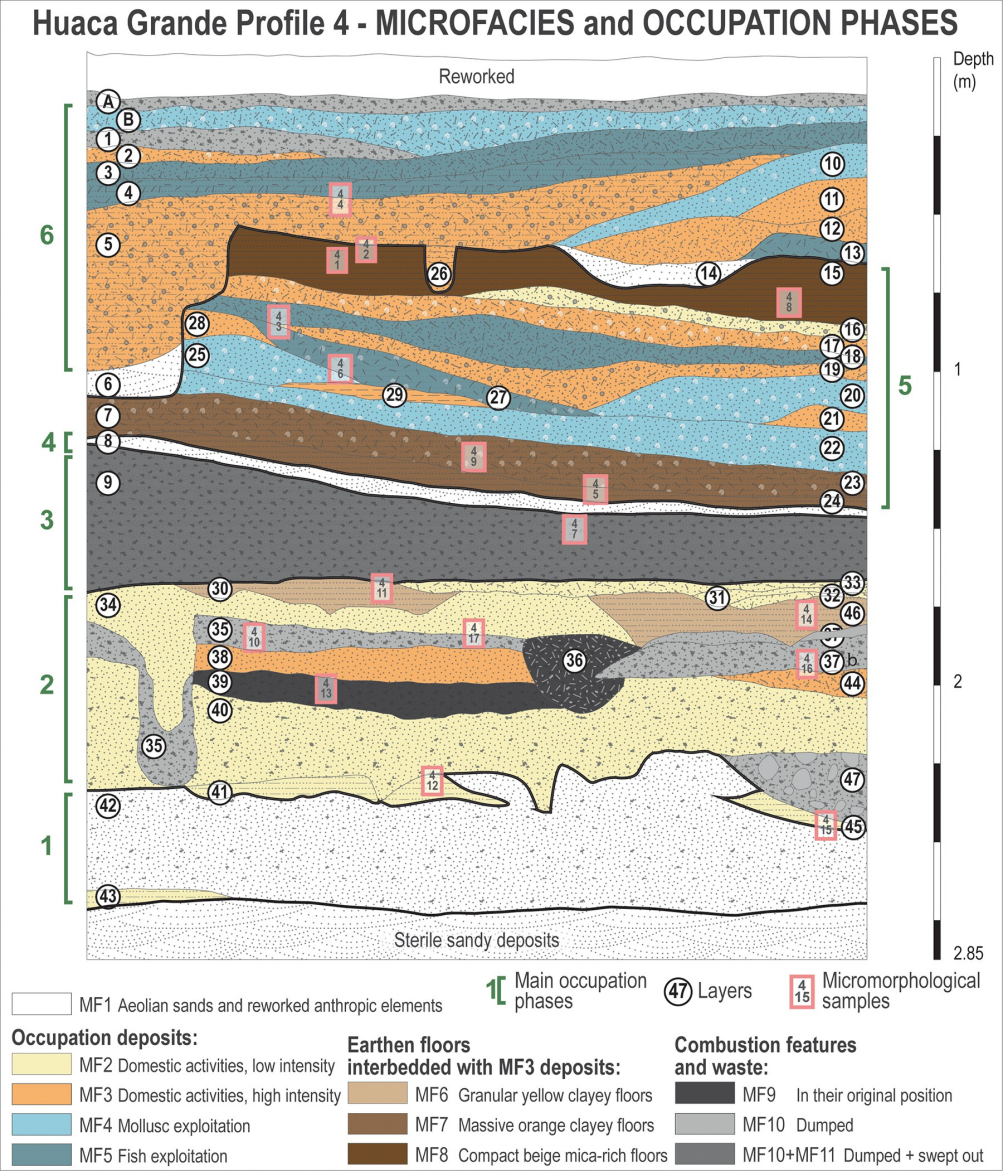
Microfacies and occupation phases distinguished along the Huaca Grande archaeological sequence.

On the basis of field observations and of our multidisciplinary study results, six main phases of human occupation of Huaca Grande can be distinguished (Table 3; Fig 13). The site was inhabited from around the first half of the 5th century CE (Fig 8). The first evidence of occupation corresponds to the building of at least two clay walls, found at the base of the sequence. The numerous post holes in the upper surface of the walls indicate that this space was partially covered. Traces of a poorly preserved yellow clay floor and of some domestic hearths are associated with these two walls, confirming that we have brought to light the inner part of the structure they belong to. It is associated with an access ramp, and the function of this structure is difficult to ascertain, as no trace of any specific activity has been preserved. It probably had a public function because of the investment in labour for the construction of the building and the ramp. Despite the presence of Mochica groups in Chusis [77], at 45 km north of Huaca Grande, we do not have evidence of their presence in the Nunura bay, around the Illescas massif and the western slope of the Sechura desert. These populations were probably in relation with the Mochicas based in Chusis, but no mochica ceramic sherd, metal artefact, textile fragment or burial were recorded during the excavations.

These architectural and functional features are covered by MF1-type deposits (layer 42), mainly constituted by aeolian sands, including some older anthropogenic elements, such as charcoal fragments, burnt fish bones, mollusc shells or herbivore excrement. These layers hence provide two different types of information. First, they attest that camelids were already present on the site and in the region during this period, as has been noted for the Chicama, Moche and Virú valleys [78, 79]. Second, they indicate that the site was abandoned at the end of this first phase of occupation. The modelled radiocarbon dates suggest that this abandonment period lasted between 0 and 117 years (46 years between the end of phase 1 and the beginning of phase 2, median dates; Fig 8).

Then, in the following period (between the end of the 6th century CE and the beginning of the 7th century CE, layers 45 to 33; Fig 8), the site was structured in a different way and characterised by an essentially domestic occupation. Some thick MF2- (45, 41, 40, 34, 31, 31 and 33) and MF3-type (38 and 44) layers with varying amounts of anthropogenic elements indicate a continuous human presence (Fig 13). Some very well-preserved, structured domestic hearths, in which several successive, distinct combustion episodes can be distinguished (MF9, layers 39 and 36), were also found. Close to these structures lay several MF10-type layers (47, 35, 37a and 37b), consisting of dumped burnt materials, resulting from their maintenance. A series of superimposed earthen floors (MF6, layers 30 to 46) associated with fairly deep post holes (about 60 cm) alternating with occupation layers (MF3) characterise the most recent occupations of this phase (Fig 13). During this period, sea mammals seem to have been one of the main resources exploited by the inhabitants of Huaca Grande for their subsistence, together with fish.

Then, the human occupation of the site is documented by a finely bedded black layer (layer 9), up to 1 m thick and consisting exclusively of reworked dumped and swept-out burnt material (MF10 and MF11; Fig 13). Macroscopic and thin-section observations indicate that the layer is not a massive deposit resulting from a single event, but, rather, a progressive accumulation, produced by the repetition of the same action over time. Part of the burnt material consists of food waste, such as mammal bone, shell and fish bone. However, most of the accumulation consists of fuel residue, in the form of charcoal and burnt plant remains, mainly two genera in the Fabaceae family (*Prosopis/Vachellia*; Fig 11). It therefore appears to result essentially from an activity requiring heating and therefore using a considerable amount of fuel. We hypothesised that the activity may have been fish smoking, such as it was possibly observed at nearby site of Bayovar-01 [76], or the production of ceramic or metal artefacts, but we did not find additional evidence to support these hypotheses. The absence of combustion structures preserved in this part of the sequence suggests that the domestic occupation or, in the case of production, the productive area, was located elsewhere, either in another sector of Huaca Grande not yet investigated or at a nearby site. This sector of Huaca Grande would therefore have functioned essentially as a dumping ground during this period.

The four radiocarbon dates performed on samples collected along layer 9 indicate that the deposition of this impressive amount of combustion residue took place over a short period, of less than one century (between 18 and 75 years according to the Bayesian modelled dates; Table 3), during the 7th century CE. According to the modelled dates, the interval between phases 2 and 3 would have lasted between 0 and 49 years (Table 3). The results of the stratigraphic and micromorphological studies allowed us to establish that the attested changes in the organisation and function of the site occurred as a continuation from the previous occupation, as no MF1-type deposits were identified between phase 2 deposits and layer 9 (Fig 13).

Thick, black deposits similar to layer 9 have been observed at the top of the archaeosedimentary sequence of some shell mounds located along the southern portion of the Brazilian coast and dated after 2500 BP [64]. One of them, Jabuticabeira II, has been the subject of multi-element chemical analyses and micromorphological analyses. These studies indicate that these huge accumulations were the result of discrete episodes of deposition of thermally altered materials burnt in a different place (35: 331). This series of anthropogenic depositional processes would have been associated with the ritual construction of a funerary mound. It is assumed that these black deposits are formed by both the residue of daily subsistence [64] and the burnt materials derived from ritual banquets and fires that took place during interments [80].

Huaca Grande layer 9 differs from the Brazilian shell mound deposits in that it is not directly associated with human burials. In its upper part, there are some animal deposits, but stratigraphic observations as well as radiocarbon dates of two of the camelids (cal. 891–1014 CE) indicate that the ritual deposits of animals post-date the formation of the black layer (layer 9). The fill of the trenches where the animals were deposited, as well as the layer (layer 8) that covers them, correspond to sterile aeolian sands (MF1-type), which extend over the entire surface of the excavation area covering the underlying black layer, layer 9. Layer 8 is very irregular in thickness, ranging from a few centimetres along the west profile (profile 4) to more than 50 cm along the east profile (profile 2). This layer testifies to a long period of abandonment of the site, which lasted about six centuries, from the mid-7th century CE to the mid-13th century CE. During this long period, human presence at Huaca Grande is only attested by this deposit of camelids and dogs.

Then, a new phase of site occupation is attested by a series of earthen floors (MF7 and MF8, layers 24, 23 and 15) alternating with activity layers (MF2-, MF3-, MF4- and MF5-types, layers 22 to 16), suggesting a continual human presence at Huaca Grande during this phase (Fig 13). The presence of small clay nodules mixed with food waste within the occupation layers, observed in thin-section, indicates that the floors were cleaned and swept regularly. The micromorphological study revealed that two different types of earthen floors can be distinguished during this phase. Both of them differ significantly from those identified in the lower part of the sequence, corresponding to more ancient levels (MF6, layer 46; Fig 13). MF7-type soils (layers 24 and 23) were prepared using clays with a different mineralogy. MF8-type soils (layer 15) are more homogeneous and massive and, unlike MF7-type soils, do not show a granular structure, despite the greater presence of the coarse fraction. In addition, they contain a much larger proportion of mica (Fig 7). Particularly interesting is the fact that within the pores and cracks of these soils, several pedofeatures (clay coatings and infillings) indicate a rather intense illuviation. This may be the consequence either of activities involving frequent use of water on these soils or of significant rainfall phenomena during this period.

**Fig 14:**
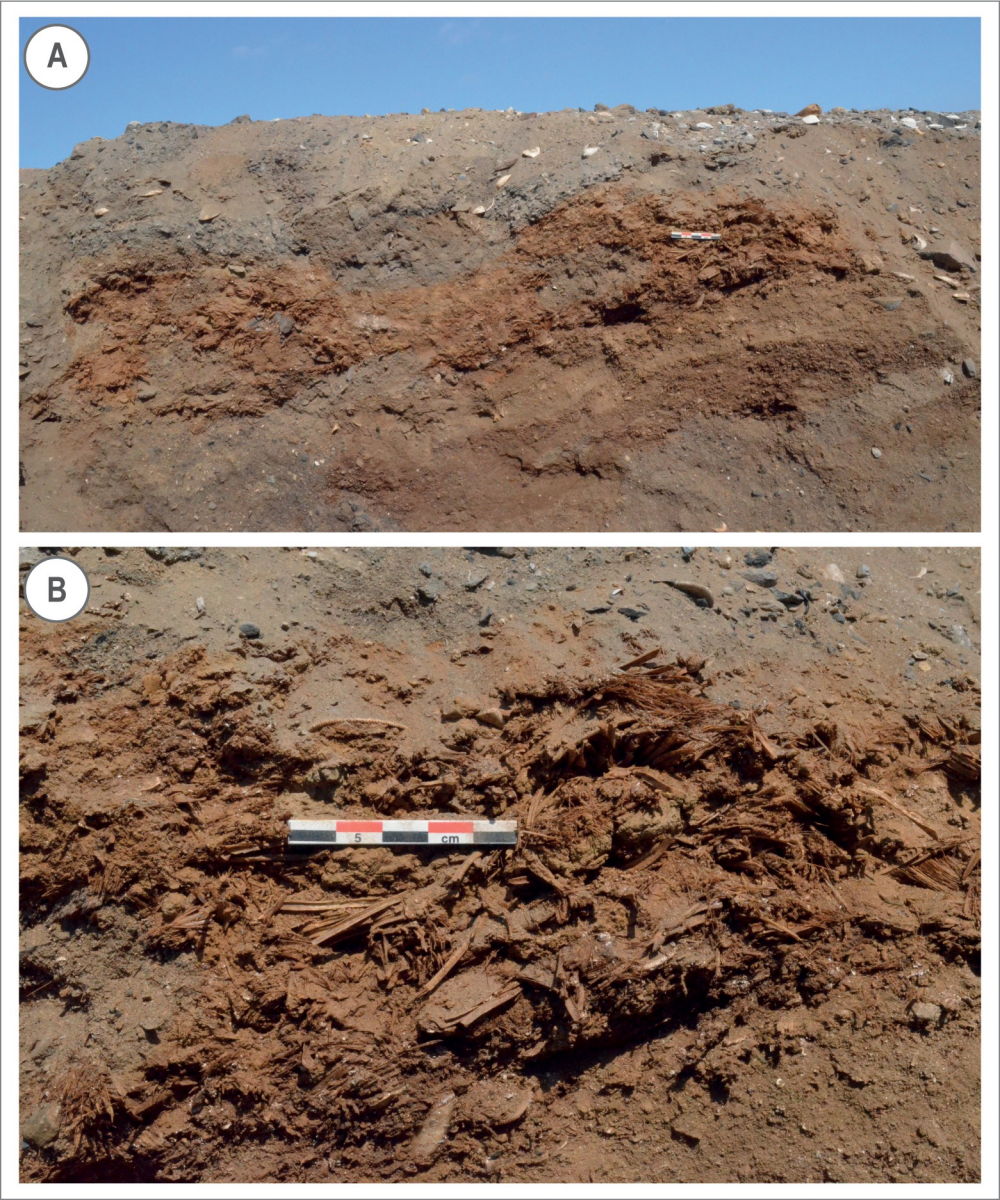
Layer 18, consisting exclusively of fish remains. A. General view. B. Close-up.

In addition to layers indicating generic domestic activities of food preparation and consumption (MF3)–and the presence of small hearths (with a diameter between about 40 and 70 cm) and their maintenance (MF10)–some facies indicating specific activities are observed here. Remains of fish and marine mollusc occur in large amounts in these facies compared with the rest of the archaeological sequence of the Huaca Grande. Fish bones and some shell debris are also present in the lower occupation levels, but they are more scattered there. Here, in contrast, some layers are mainly made up of shell (MF4, layers 22, 20 and 25), fish remains (MF5, layers 27 and 18), or a mixture of both (MF3, layers 29, 21, 19 and 17) and therefore attest to regular catching of fish and gathering of molluscs and their consumption on-site. These activities seem to have replaced mammal hunting as the main component of the subsistence economy. Among these deposits, a concentration of fish remains localised in layer 18 is particularly interesting because it consists exclusively of *Scomber japonicus* gills (Fig 14).

This indicates that intensive fishing activities took place and that fish were prepared on-site, probably by removing the heads, viscera and gills, either for immediate consumption or for preservation for later consumption, after smoking or salting. Considering layer 18’s very reduced thickness, the fact that all the remains within it belong to the same species, and its uniqueness within the sequence, its deposition can be considered as an isolated episode. This suggests the absence of a fixed subsistence economy based on a specific resource.

The study of the malacological and other marine invertebrate assemblages from Huaca Grande supports this hypothesis. In fact, the three most-consumed species (*Donax obesulus*, *Acanthopleura echinata* and *Argopecten purpuratus*) in terms of NISP share the same vertical distribution (mesolittoral and infralittoral) but not the same habitat (sandy vs rocky). This indicates that they were not the product of a specialised gathering activity. Similarly, there was no systematic exploitation of multiple areas or biotopes; only the nearshore species that were most easily found on the sandy beaches and rocky outcrops near Nunura Bay were collected (Fig 10).

As for camelids, their presence at the site during all phases is indicated by excrement occurring throughout the stratigraphic sequence. On the other hand, the absence of a large quantity of bone remains, with the exception of the ritual deposit identified in the central part of the sequence (top of layer 9), suggests that camelids were not used for consumption but that their function was primarily related to trade activities.

This period of occupation lasted until the beginning of the 14th century CE. It ended with a series of pits that cut deep into the underlying layers (Fig 13). They most likely contained large ceramic vessels (*tinajas*), which were probably used for storage. The fill of these pits consists of a sterile grey sandy sediment (MF1), identical to that of layer 8. It therefore seems to attest to a new phase of abandonment of the site, most probably of short duration, as its thickness never exceeds 10 cm. Modelled radiocarbon dates are coherent with this hypothesis, as they suggest that the abandonment lasted about 70 years (between 0 and 136 years; Table 3). This period corresponds chronologically to the Chimú period but apart from the burials found by Cárdenas et al. [1] and our team, no archaeological artefact characteristics of this culture was found during the excavations. The presence of Chimús is not permanent but looks more like a periodic occupation since no architectural structure was recorded in the top of sequence. The occupation, in perishable materials as attested by several post holes, is more temporary and probably linked to subsistence strategies.

The archaeological stratigraphic sequence ends with another succession of occupation layers (MF3, layers 12, 11, 5 and 2) and accumulations of shells (MF4, layer B) and fish remains (MF5, layers 13, 4 and 3) alternating with reworked combustion residue (MF10, layers 1 and A). The subsistence economy during this period appears to have been similar to that of the previous occupation phase, based on fishing and shellfish gathering and supplemented with mammals, albeit with a reduced presence of mammals.

Some remains of economically valuable plants have been found, most notably in the upper part of the sequence (layers 8 to B). These plants are primarily maize, a food staple, and cotton, central to various crafts, such as the making of cloth or fishing nets. Cucurbitaceae are also of economic importance, either as food, such as squash (*Cucurbita* sp.), or as craft material, such as gourds (*Lagenaria* sp.). Soapberry fruits also have a varied array of possible uses (craft, cleaner, medicinal). None of these plants would have easily grown in numbers in the Nunura Bay area without at least some kind of irrigation and management. It is more likely that they arrived at the site through exchange or trade networks, which seem to have been more intense during the last phases of occupation. It also seems that craft activities using cotton became more important at the time. The most recent date for these occupations ascribes them to the middle of the 15th century CE, and this matches the Chimú-Inca-period date of the two human burials found in area A4.3 (Fig 5).

### Palaeoenvironments

Zooarchaeological and archaeobotanical analyses provide information not only on the subsistence economies and natural resources exploited by the human groups that settled at Huaca Grande, but also on changes in the environments around the site. Even though they provide only a partial framework, as only species and taxa chosen and linked to human activities are present, they yield clues which, when cross-referenced, offer an overview of coastal environments over time. They also permit us to identify some intense climatic events that may have taken place during the history of the site.

The malacological study does not show any major climatic or palaeoenvironmental change during the Huaca Grande occupation and indicates that the environment around the site was very similar to the present one. The vast majority of the molluscs species identified at Huaca Grande, particularly the three most-consumed species (*Donax obesulus*, *Acanthopleura echinata* and *Argopecten purpuratus*), are present today in the area surrounding the site. The presence of both temperate-water and tropical species (Fig 9) is consistent with the geographical location of the bay, on the southern border of the Paita Transition Zone. This is a dynamic environment, and the distribution limits of some mollusc species vary according to sea temperature. Therefore, the presence of tropical species is not necessarily the consequence of a climatic anomaly associated with an El Niño event, because some species, such as *Cerithium muscarum* and *Iliochione subrugosa*, can also live in lagoon or mangrove environments. These two species, identified in very low proportions at Huaca Grande, have previously been registered in the Las Salinas palaeolagoon [16]. Moreover, during El Niño events, the demography of some species, such as *Thaisella chocolata* and *Argopecten purpuratus*, is positively affected, resulting in a strong biomass increase [81, 82]. However, the malacological assemblages from Huaca Grande did not record an increase in these species. The occurrence of the above-mentioned tropical water species could be due to transient increases in sea temperature associated with low-amplitude El Niño events. However, it is important to point out that the total absence of the species *Mesodesma donacium* from the Huaca Grande malacological record is consistent with the other evidence of fairly frequent El Niño events. *M*. *donacium* is a species that inhabits both intertidal and subtidal environments and occupies the same ecological niche as *Donax obesulus* [83]. However, unlike *D*. *obesulus*, *M*. *donacium* is particularly sensitive to changes in water temperature, and its populations decrease drastically and disappear during increases in SST caused by El Niño episodes [84]. Research by Sandweiss et al. [11, 28, 85] on malacological assemblages from several archaeological sites on the Peruvian coast shows that *M*. *donacium* is one of the most intensively economically exploited species since the end of the Pleistocene due to its extremely high biomass. Its presence in the shell middens of the northern sites is, however, discontinuous; indeed, it is present only under conditions of diminished frequency of El Niño events and disappears, replaced by the much smaller *D*. *obesulus*, when El Niño events occur with a frequency comparable to the present. Thus, its complete absence at Huaca Grande indicates that during the period of use of the site, El Niño events occur with sufficient amplitude and frequency to prevent this species from proliferating in the marine environments of Nunura Bay.

In the same way, the fish identified are temperate-water species. This fact could be considered surprising, at least for the bottom of the Huaca Grande sequence, which corresponds in time to the occupation of the site of Bayovar-01 (5th–8th centuries CE). At this fishing site located at the edge of the Las Salinas palaeolagoon, in the Reventazón area, 35 km southeast of Nunura Bay, warm-water species, such as *Micropogonias altipinnis* and *Albula* sp., were registered associated with temperate-water species, such as *Mugil cephalus* [4, 5]. It is also possible that El Niño events contemporary with the occupation of the site did not cause major changes in the marine environments of Nunura Bay, or that their impacts on these environments were not sufficiently intense and/or long-lasting to cause a drastic shift in species distribution than in the Reventazón area.

The anthracological and carpological studies show that almost all of the identified taxa are still present in the area surrounding Huaca Grande today. *Sesuvium portulacastrum* and *Batis maritima* are halophytic succulent shrubs commonly found on the coast near the ocean shore, as well as in salt or brackish marshes, salt-pans and mangroves [86–88], and they can be found on the beach at Nunura Bay today. *Tiquilia paronychioides* is a common desert shrub in coastal northern Peru, where it grows on sand or sandy clay [89]; it is present today in the *quebradas* surrounding Huaca Grande.

*Prospis* sp., *B*. *avicenniifolia*, *C*. *scabridum*, *P*. *aculeata* and *S*. *spicata* are xerophytic trees and shrubs characteristic of the dry tropical forest ecological formation of the northern coast of Peru [90]. Among the components of this ecological formation, these taxa can grow where hydric resources are least available. All of them are present today around Huaca Grande. *Prosopis* sp. is the more abundant taxon, especially along the *quebradas*, whereas *B*. *avicennifolia* and *C*. *scabridum* inhabit the nearby terraces. The rarest is *P*. *aculeata*, which is only found in the *quebradas*. *Prosopis/Vachellia* is by far the most ubiquitous taxon throughout the archaeological sequence. This can be easily explained by the properties of its wood, being very dense and slow burning, which makes it a very good source of fuel. If available, a combination of *Prosopis* sp. and *V*. *macracantha* usually was the primary firewood used by pre-Hispanic societies on the north Peruvian coast [53, 91]. The predominance of this taxon in the anthracological spectrum (as well as the quantity of the recovered material) suggests that this particular firewood source was easily available during all the occupations of the site.

*Maytenus octogona* and *Cryptocarpus pyriformis* are also common xerophytic shrubs of the dry tropical forest. They can be found in areas with a little more humidity, such as desert margins, including the Illescas massif, where they have been found and described (70: 277–278). Nonetheless, neither is present in the vicinity of Nunura Bay nowadays. The presence, although incidental, of *C*. *pyriformis* wood in the lower half of the sequence (layers 42-L, 40, 35, 35-F1 and 9-L), could reflect less arid conditions around Huaca Grande at the time. However, wood from this shrub could have been gathered farther inland, on the Illescas massif, where it grows today, and brought to the site. Alternatively, it could have been carried down from the Illescas massif by violent floods, like those associated with El Niño episodes; its presence could thus be a marker for this kind of event.

The *Prosopis/Vachellia* and Fabaceae proportions decrease significantly only at the end of the occupation sequence (layers A and B). This could be related to (i) a particular activity being carried out using different firewood assemblages; (ii) a change in preservation conditions; or (iii) a decline in the availability of this resource, as a result of either previous overexploitation or a change in environmental conditions. The same hypotheses can be advanced to explain the presence of *C*. *scabridum* at the top of the sequence. This shrub is very often found along with *Prosopis* sp. and is found by itself only in the interior of the desert [92, 93]. This could hence reflect an opening/clearing of the vegetation favouring the presence of *C*. *scabridum* over *Prosopis* sp.

The presence of *H*. *versicolor* in layer 4 is consistent with this hypothesis. This Cactaceae is known to be present in the dry tropical forests of the Piura and Lambayeque regions [93, 94], although it is not present in the vicinity of the Nunura Bay beach today. Its presence could reflect dryer conditions at this time that could relate to the clearing of the dry forest–type vegetation. Or the seeds and/or fruits could have been brought from another area as a result of exchange. However, the presence of *M*. *octogona* in the same part of the sequence (layer B) would argue against this idea, as it is found in denser and more diverse vegetation. Its presence remains incidental, and it could be the result of a supply from farther away and therefore not reflect local conditions. As explained above, a particular set of activities requiring the use of *C*. *scabridum* wood could also explain this occurrence. The presence of Fabaceae and *Prosopis/Vachellia* in high proportions together with that of *C*. *scabridum* in layers 18 and 8 would argue against the preservation hypothesis, according to which the increased proportions of the latter are the result of different preservation conditions.

Only two taxa, found incidentally at the base of the sequence (layers 41 and 42-L, respectively; Fig 11) are completely foreign to the Nunura and Illescas environments and are not common constituents of the dry tropical forest. *Muntingia calabura* grows on disturbed sites in tropical lowlands but is intolerant of saline conditions [95]. It can be found in the dry tropical forest, but also in denser and more inland formations, such as the Bosque Pomac (160 km southeast of Huaca Grande, in the La Leche valley) [92, 96]. *Salix humboldtiana* grows mainly along river courses and in humid soils. In Piura, it was listed near the main rivers, the Piura and the Chira, and it was also found in the Pomac forest [92].

Neither of these two taxa could naturally have been present near Huaca Grande. It is possible that some trees were carried from the interior to the coast by violent floods. As neither of them have been found on the Illescas massif (where all temporary watercourses emptying in Nunura Bay originate), it is more likely that specimens of these trees came from river valleys farther away, such as the Piura valley. Carried by floods and then deposited on the beach as driftwood, they could have been picked up and used, possibly as firewood. Lastly, it is also possible that wood from these trees was intentionally brought to the area by human groups for specific uses.

### Correlation between site occupations and changes in climate

Further discussion and interpretation of the results on occupation pattern, change in the resources exploited and change in the environments around the site require a comparison with the regional palaeoclimatic record. The occupation of the site corresponds to a period during which the average climate was affected by several variations: i) a wet phase characterised by a high frequency of extreme El Niño events, comparable in intensity to those of 1982/1983 and 1997/1998, which lasted until the middle of the 8th century CE and which is recorded at the base of the sequence, then ii) a phase marked by more arid conditions, which coincides with a weakening of El Niño between 750 and 1250 CE; and, finally, iii) a return to a more humid climate with a resumption of El Niño activity after 1250 CE [31, 97].

Despite several radiocarbon ages that constrain the timing of successive occupation phases at Huaca Grande, the chronological resolution available is not precise enough to establish a clear cause-and-effect link between high-frequency short climatic oscillations (including El Niño events) and periods of human frequentation, successive reorganisations of the site and changes in its function. However, stratigraphical and archaeobotanical data allow us to make some hypotheses.

Indeed, although the botanical remains attest to a certain stability of the environments around the site over the span of a millennium, multiple clues indicate more occasional, abrupt variations in humidity, probably linked to El Niño-related flooding. In the upper half of the sequence, whose formation occurred from the 7th century CE according to radiocarbon dating, the vast amount of *S*. *portulacastrum* remains in layer 9 (9-U, 9-M and 9-L) and especially in layer 35 could result from particular activities involving this plant taking place at that time. However, it could also indicate a larger presence/availability of the plant in the vicinity of the site resulting from environmental changes in Nunura Bay that favoured its growth. One such change could have been the presence or expansion of bodies of salt or brackish water, perhaps related to El Niño floods.

The NISP of *T*. *paronychioides* also significantly increases in the middle and upper parts of layer 9. This could result from wetter and more favourable conditions, possibly due to flooding, than existed during the formation of the lower part of the section and layer 35, where this species is rare. This hypothesis seems to be confirmed by the presence of *E*. *maritimus*, which follows the same trend. This Solanaceae is known to rapidly expand in the desert after rain events such as those associated with El Niño and to then die out [98]. We personally observed this phenomenon on the Huaca Grande mound and in its vicinity just after the 2017 event [7]. This species’ presence in significantly higher numbers in layer 9 (9-M and 9-U) likely reflects the aftermath of one or more rain events (probably related to EN) at the time this layer was forming. It is likely that the site was abandoned due to one of these extreme events.

Throughout the following arid phase, between the middle of the 8th century CE and the middle of the 13th century CE, no permanent occupation took place at the site, and the only traces of human presence relate to the deposition of several animals. This indicates that human groups were still frequenting the bay, but that during this period, it probably did not offer favourable conditions for continuous settlement. Those who deposited the animals were perhaps the human groups (Lambayeque-Sicán culture) living at the nearby site of Huaca Amarilla [6] or other groups moving around the Sechura Desert.

Huaca Grande was occupied once again when the climate became wetter, in the middle of the 13th century CE. This settlement phase lasted more or less continuously for two centuries (remember the short abandonment attested by layers 6 and 14, around the beginning of the 15th century CE). We can suggest that these populations were linked more strongly with the Huaca Amarilla people and that they seem to be different from the populations of the older occupations. Indeed, the presence of incised ceramics at the bottom of the sequence and of *paleteado* ceramics and Chimú-Inca type ceramics in the two burials at the top permit to distinguish at least three cultural traditions. The subsistence economies are also different: the earliest group occupying the site used more sea mammals and the latest ones used more molluscs and fish. This pattern is quite different from the groups who settled at Huaca Amarilla, who had access to camelids for their diet, probably due to the occupants of Huaca Amarilla having a higher status. The architecture made of two stone structures, a large domestic area, architecture with coated walls, evidences of storage, burials show that the site of Huaca Amarilla was a civico-ceremonial center of Lambayeque-Sicán culture [6] then occupied by Chimús and Chimús-Inkas groups.

Overall, we can suggest that the phases of wetter average climate and rather intense El Nino activity, up to the middle of the 8th century CE and after 1250 CE, would have allowed for an almost continuous human presence on Nunura Bay, as the local environments would have provided sufficient plant and marine resources to sustain the groups settled on the site. Instead, the arid period between 750 and 1250 CE would have been less favourable for the permanent settlement of human groups in Nunura.

## Conclusion

The integrated study of the Huaca Grande sequence has permitted us to characterise the occupation of this multifunctional mound over the span of a millennium. Although it has often been identified as a domestic mound, we observed that Huaca Grande did not have a uniform history. It was used successively in several ways, as a domestic, ceremonial, and funerary place. The first human groups who settled on this site, associated with clay public architecture, had an economy turned towards the sea and took advantage of favourable climatic conditions. However, for largely unknown reasons, but partly related to the advent of more arid climatic conditions, the site was abandoned for five centuries. Only the deposition of camelids and dogs interrupted this abandonment. This deposit was a very short-time event and corresponds to some kind of foundation or abandonment offering. In this case, we can suggest that we faced to an event probably linked to the installation of Lambayeque-Sicán nearby site of Huaca Amarilla. Indeed, the chronology of the deposit is coherent with the beginning of Huaca Amarilla occupation around the IX-Xth centuries CE. During the second phase of occupation of Huaca Grande, its inhabitants continue the pattern of exploiting the local natural resources, they acquired other goods (maize and cotton) by exchange, showing that the site had acquired a higher status. The presence of these two taxa is probably linked to the activities that take place in Huaca Amarilla. In this site, 71 burials (70 of immature and one adult) were found and some of which had funerary goods composed by vessels, necklaces and bracelets, and other wood figurine [99]. Some of the component of these goods, particularly the necklaces and bracelets, were imported such as turquoise, chrysocolla, sodalite or Spondylus. During the abandonment of Huaca Grande, populations occupied Huaca Amarilla and built some important structures in this place. When the permanent occupation of Huaca Amarilla is abandoned, we observed the re-occupation of Huaca Grande. Thus, we observed different layers of a domestic occupations while in Huaca Amarilla, the site is only use for funerary purposes. In consequence, the relation between the two site is quite difficult to determine but show complex exchanges with the presence in both sites of special goods and special burials. At the end of that occupation, Huaca Grande once again experienced a change and became a funerary sector. The multidisciplinary approach we developed for this site shows not only the capacity by human groups peopling the Sechura Desert over a period of 1000 years to adapt to this arid environment, but also their response when faced with a major climatic event, such as El Niño.

## Supporting information

S1 Fig Location of the three excavation areas on the Huaca Grande mound. (TIF)

S1 Table Huaca Grande archaeological sequence. Detailed description of the stratigraphic layers.
(XLSX)

S1 Appendix Huaca Grande Bayesian model, OxCal codes. (DOCX)
