# Supplementary figures and images for: Settlement dynamics, subsistence economies and climate change during the late Holocene at Nunura Bay (Sechura Desert, Peru): A multiproxy approach
Source: PLoS One. 2023 Mar 9;18(3):e0281545. doi: 10.1371/journal.pone.0281545 (PMC9997921; doi:10.1371/journal.pone.0281545)

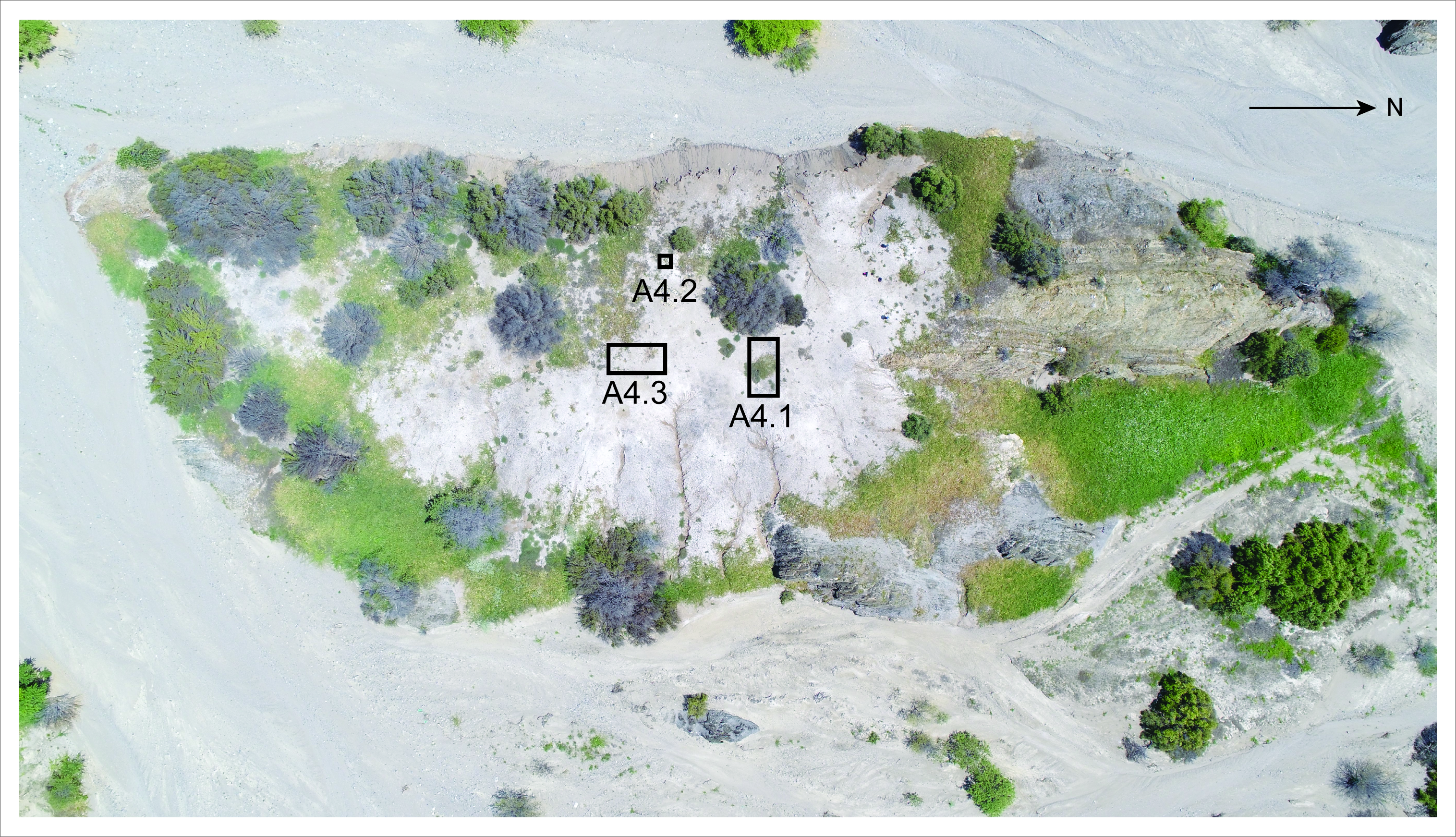

Supplement: S1 Fig — (TIF) [file pone.0281545.s001.tif]
