# Supplementary material for: Settlement dynamics, subsistence economies and climate change during the late Holocene at Nunura Bay (Sechura Desert, Peru): A multiproxy approach
Source: PLoS One. 2023 Mar 9;18(3):e0281545. doi: 10.1371/journal.pone.0281545 (PMC9997921; doi:10.1371/journal.pone.0281545)
Supplement: S1 Appendix — (DOCX) [file pone.0281545.s003.docx]

**S3 Appendix. Huaca Grande Bayesian model, OxCal codes**

Plot()

{

Curve("SHCal20","shcal20.14c");

Sequence("Huaca Grande")

{

Boundary("Phase 1 Start");

Phase("Phase 1")

{

R_Date("UBA-30844-Camelids faeces", 1602, 29);

Span("Span of Phase 1");

Interval("Duration Phase 1");

};

Boundary("Phase 1 End");

Interval("between Phase 1 and Phase 2");

Boundary("Phase 2 Start");

Phase("Phase 2")

{

R_Date("UBA-41157-Charcoal", 1471, 25);

R_Date("UBA-35567-Charcoal?", 1547, 26);

R_Date("UBA-41158-Camelids faeces", 1547, 22);

Span("Span of Phase 2");

Interval("Duration Phase 2");

};

Boundary("Phase 2 End");

Interval("between Phase 2 and Phase 3");

Boundary("Phase 3 Start");

Phase("Phase 3")

{

R_Date("UBA-35566-Seeds", 1506, 28);

R_Date("UBA-35565-Camelids faeces", 1467, 24);

R_Date("Beta-454075-Charcoal", 1430, 30);

R_Date("UBA-35564-Charcoal?", 1421, 29);

Span("Span of Phase 3");

Interval("Duration Phase 3");

};

Boundary("Phase 3 End");

Interval("between Phase 3 and Phase 4");

Boundary("Phase 4 Start");

Phase("Phase 4")

{

R_Date("ECHo 2998-bone collagen", 1148, 26);

R_Date("ECHo 2999-bone collagen", 1144, 26);

Span("Span of Phase 4");

Interval("Duration Phase 4");

};

Boundary("Phase 4 End");

Interval("between Phase 4 and Phase 5");

Boundary("Phase 5 Start");

Phase("Phase 5")

{

R_Date("UBA-30843-Camelids faeces", 796, 28);

R_Date("UBA-41156-Camelids faeces", 755, 19);

Span("Span of Phase 5");

Interval("Duration Phase 5");

};

Boundary("Phase 5 End");

Interval("between Phase 5 and Phase 6");

Boundary("Phase 6 Start");

Phase("Phase 6")

{

R_Date("UBA-35563-Seeds", 546, 31);

Span("Span of Phase 6");

Interval("Duration Phase 6");

};

Boundary("Phase 6 End");

};

};
